# Measurement of critical health literacy in primary school pupils: a Polish validation of the Claim Evaluation Tools

**DOI:** 10.1136/bmjopen-2025-099994

**Published:** 2025-07-17

**Authors:** Anna Prokop-Dorner, Katarzyna Zawisza, Maria Świątkiewicz-Mośny, Aleksandra Kobla-Piłat, Natalia Ożegalska-Łukasik, Magdalena Ślusarczyk, Aleksandra Potysz-Rzyman, Malgorzata M Bala

**Affiliations:** 1 Department of Medical Sociology, Chair of Epidemiology and Preventive Medicine, Jagiellonian University Medical College, Kraków, Poland; 2 Institute of Sociology, Jagiellonian University, Krakow, Poland; 3 Institute of Intercultural Studies, Jagiellonian University, Kraków, Poland; 4 Department of Hygiene and Dietetics, Chair of Epidemiology and Preventive Medicine, Jagiellonian University Medical College, Kraków, Poland

**Keywords:** Health Literacy, Health Education, Adolescents

## Abstract

**Abstract:**

**Objectives:**

Amid increasing adolescents’ immersion in media information and their vulnerability to mis/disinformation and the screen time negative health correlates, competences of critical thinking about health gains in significance. The Claim Evaluation Tools (CET) assess the understanding of claims about treatment effects and enable critical health literacy measurement in different populations. It has not been adapted and tested in Central Europe before.

**Design:**

The process of cultural adaptation covered three phases: translation and cultural adaptation, testing and psychometric analysis. We used Classical Test Theory and Rasch analysis to verify the validity and reliability of the Polish version of the CET. Additionally, we tested known-group and convergent validity.

**Setting:**

The pilot test was conducted on a pupils sample from eight purposefully selected schools in Southern Poland, and the final version of the tool has been tested on a national study sample of pupils of primary schools randomly selected from all Polish regions.

**Participants:**

We collected the data from a national sample of 2242 pupils aged 12 to 15 years, attending sixth to eighth grades, from 42 primary schools.

**Results:**

We applied some changes to the multiple-choice questions (MCQ) scenarios and wording of some of the options based on the expert’s opinions. We validated 24 MCQs reflecting 12 claims about treatment effects (2 MCQs per claim). The psychometric properties of the Polish version of the tool are satisfying.

**Conclusions:**

The Polish version of the CET can be applied to measure critical health literacy among 12- to 15-year-old adolescents as well as to evaluate educational interventions promoting the understanding of healthcare claims in Poland.

**STRENGTHS AND LIMITATIONS OF THIS STUDY**

- To ensure a comprehensive approach to cultural adaptation, the processes involved the interdisciplinary team of experts and researchers.
- The clarity of the translated items was verified based on experts’ and pupils’ feedback.
- We applied Classical Test Theory and the Rasch analysis to validate the Polish version of the Claim Evaluation Tools.
- A limitation of the study is that participants’ health status was not verified, which prevented subgroup analyses comparing children with and without health problems.

## Introduction

 In the face of digital usage becoming one of the most time-consuming activities in children’s and adolescents’ daytime,^1^ their vulnerability to mis/disinformation^2^ and the screen time negative health correlates,^3 4^ the competences of critical thinking are gaining even greater significance than before.

The studies conducted in various cultural contexts suggest that adolescents want to receive health information through social media,^5 6^ share and forward health information through online channels and trust especially information posted by peers.^7^ The ability to navigate through health information available in media requires, among others, the competences of critical health literacy (CHL) and the ability to assess health claims.

Health literacy is defined as knowledge, motivation and competences to access, understand, appraise and apply health information in order to make judgements and take decisions concerning health. It covers functional health literacy, interactive health literacy and CHL.^8^ For adolescents, CHL is particularly important as it encompasses the ability to obtain, understand and critically appraise different sources of information, while also engaging in shared decision-making.^9^ Given adolescents’ developmental stage, CHL supports their capacity to navigate health information critically, which is essential in an age of extensive digital media exposure and peer influence.

The initiative Informed Health Choices (IHC), developed by the IHC network, is based on the CHL conceptualised as the ability to critically appraise information about the effects of particular actions and to make informed health choices and is connected to the IHC Key Concepts educational interventions for children and adults.^10^ The IHC Key Concepts are the principles for evaluating trustworthiness of claims about effects of treatments (concepts about claims), comparisons of treatments (concepts about comparisons), as well as for making well-informed choices about treatments (concepts about choices). Key Concepts have been iteratively generated based on review of literature and critical appraisal tools for the public and professionals.^11^ The list of Key Concepts has been revised four times. Currently, the Key Concepts list includes a total of 49 health claims and is organised in 3 main groups (claims, comparisons, choices) and 10 subgroups.^12^ Subsequently, the IHC network developed the Claim Evaluation Tools (CET) database to evaluate people’s ability to assess claims about treatment effects.^10^ The CET is an open-source battery of 188 multiple-choice questions (MCQs) measuring the ability to apply knowledge, interpret and judge the information about claims reflecting one of the Key Concepts. Each of the Key Concepts is illustrated by three or four MCQs in the database. Each of the MCQs presents a scenario leading to a treatment claim and a question with three or four options. The authors developed all MCQs with a continuum of options: one option being the ‘best’ and the remaining options being ‘worse’.^13^ For teaching children in primary schools, the authors indicated 12 Key Concepts selected by primary school teachers using a modified Delphi technique, as of the most relevant and easily comprehensible for primary school pupils.^14^ Among those 12 Key Concepts, 8 regard recognising unreliable claims, 3 recognising reliable evidence and 1 making well-informed choices (table 1).

**Table 1:** 12 IHC Key Concepts for primary school pupils with labels from educational materials

Concepts about claims	Concepts about comparisons	Concepts about choices
Do not assume that treatments are safe (100% safe!) Do not assume that comparisons are not needed (No comparison needed!) Do not assume that a single study is sufficient (A study shows!) Do not assume that a treatment is helpful or safe based on how widely used it is or has been (Old is better!) Do not assume that a treatment is better based on how new or technologically impressive it is (New is better!) Do not assume that there are no competing interests (As advertised!) (It worked for me!) Do not assume that opinions alone are sufficient (Recommended by expert!)	Consider whether the people being compared were similar (Dissimilar comparison groups) Consider whether the people being compared knew which treatments they received (Dissimilar expectations) Be cautious of small studies (Few people or events)	Weigh the benefits and savings against the harms and costs of acting or not (Do the advantages outweigh the disadvantages?)

Prepared based on the publication of the IHC network.^12^

IHC, Informed Health Choices.

The iterative process of the CET development was conducted by researchers from six high- and low-income countries from three continents. It included the following phases: development of items, expert assessment, assessment of relevance, understanding and acceptability among pupils, parents, teachers and patients and finally piloting in primary school pupils in four countries.^10^ The CET has been validated using Rasch analysis.^15^ It was found to have satisfactory reliability, unidimensionality in the data and a high level of item difficulty.^10 13^ The CET has been initially designed to measure primary outcome in the IHC project’s randomised trials and evaluate CHL in other contexts. Due to its structure, the CET allows educators to tailor the tool to the teaching goals and the Key Concepts selected to best fit the needs of the learners.^10^

The tool was initially created in English and then has been contextualised and validated in Uganda,^15^ Mexico,^16^ China,^17^ Croatia,^18^ Germany and Austria,^19^ in primary and secondary schools as well as in adults.^20 21^ To our knowledge, it is the first study to adapt the CET in Central Europe, which aligns with the Visegrád Group, comprised of Czech Republic, Poland, Slovakia and Hungary, sharing historical, social and cultural background. So far, the available data on health literacy in children in this region have been collected only using subjective measures.^22^ The aim of our study was to adapt and validate the CET for the Polish cultural context of sixth to eighth grade primary school pupils aged 12 to 15 years. Our preliminary results were presented before.^23^

## Material and methods

**Figure 1:**
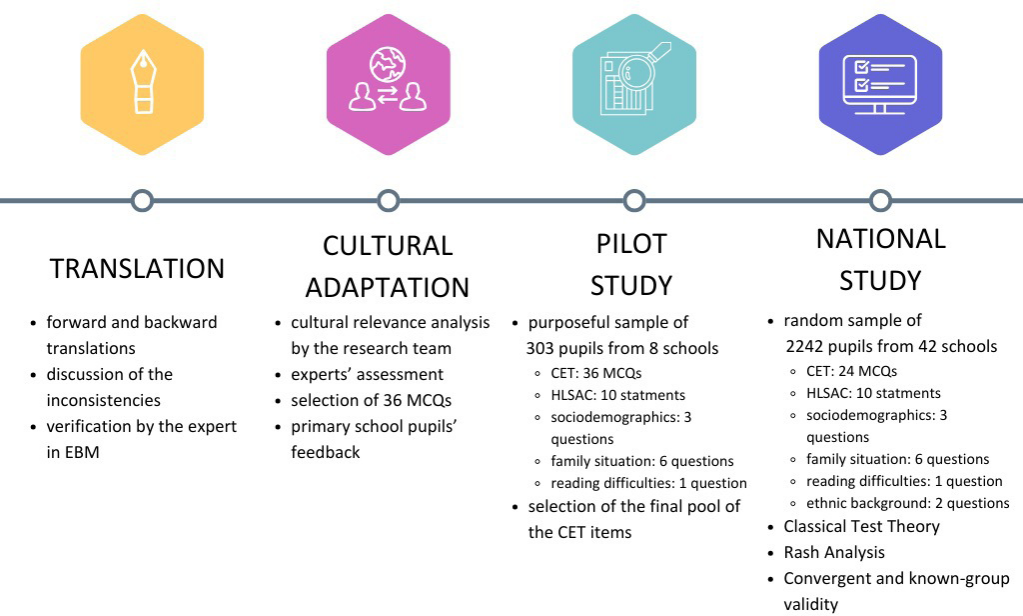
Phases in the Claim Evaluation Tools adaptation and validation process. CET, Claim Evaluation Tools; EBM, evidence-based medicine; MCQ, multiple-choice question; HLSAC, Health Literacy for School-Aged Children.

The process of the adaptation and validation comprised three phases: translation and cultural adaptation, pilot testing and cross-sectional national survey. The results from the national study enabled us to verify the psychometric properties of the Polish version of the CET (figure 1).

### Translation and cultural adaptation

Two independent translators with experience in health-related translations prepared forward and backward translations of the CET database. The original version of the CET and the backward translation were compared, and the inconsistencies were discussed in the interdisciplinary research team. Next, the expert in evidence-based medicine verified the translation in regard to correctness of terminology and plain language.^13^ Finally, seven researchers independently ranked the cultural relevance of MCQs recommended to primary school pupils,^14^ taking into account adequacy of cultural context. Based on the ranking, from the translated base of MCQs, the three highest MCQs reflecting each of the 12 Key Concepts were selected to be further assessed by experts.

We purposefully sampled three external experts based on their experience in teaching health-related content in primary schools. In the expert evaluation participated a health psychologist, a biology teacher and a school counsellor. Experts were presented with 36 MCQs together with the explanation of each of the claims. They used 4-point scales to independently evaluate each MCQ in regard to: the difficulty relevance for the age group, clarity and the readability and cultural relevance. Moreover, the experts assessed whether each MCQ covered the concept it addressed, suggested improvements and ranked the questions. Based on the expert assessment and feedback, we adjusted 36 MCQs, namely we adjusted the wording of the scenarios and response options to the 12- to 15-year-old pupils’ needs and capacities, especially in regard to plain language used to describe health problems. The adjustments included (1) names used in the scenarios to more recognisable ones in the Polish context; (2) health practices more relatable to the Polish context across all social groups (eg, eating chocolate instead of mango; chewing gums for healthy teeth instead of eating bananas). Additionally, we excluded those MCQs which reflected practices unfamiliar in the studied context, presented content unrelatable to primary school pupils or treatments less popular these days among the youth, for example, using herbal therapy.^20^ Finally, a convenience sample of fifteen adolescents aged 12 to 15 years filled the questionnaire and provided their comments.

### Pilot study in schools

The pilot-testing survey was conducted in March 2023 in eight schools in Southern Poland: two in large cities (more than 120 000 citizens), two in small towns (between 25 000 and 120 000 citizens) and four from rural areas. We estimated the sample size based on the number of items included and on the number of the personal factors we intended to explore for Differential Item Functioning in the Rasch analysis.^13^

We selected 12- to 15-year-old pupils from sixth to eighth grade of the sampled primary schools. We obtained written informed consent both from pupils and their parents and trained moderators to collect the data. Pupils during their school hours were seated in a computer room. Moderators instructed pupils how to fill in a questionnaire and about the time allocated to completing the survey (40 min). Response to every question was automatically required, to prevent missing data. After all pupils completed the test, the moderator inquired about their perception of the test.

The response to each of 36 MCQs was scored as correct (1 point) or incorrect (0 points). Additionally, we asked 3 questions regarding socio-demographics (gender, age, school grade), 6 regarding family situation and family’s socioeconomic status, 1 regarding self-reported reading difficulties and used the Health Literacy for School-Aged Children (HLSAC-10, 10 questions).^24^ The HLSAC-10 measures subjective HL on five dimensions: theoretical knowledge, practical knowledge, critical thinking, self-awareness, citizenship.^24^ Respondents respond to 10 statements on a four-level scale: not at all true, not quite true, somewhat true, absolutely true. The tool has been validated in several European countries, including Poland.^25^ As the HLSAC-10 has been proven reliable on a sample of Polish teenagers, we used it to assess the construct of the CET.

Moreover, we used the results from the pilot study to establish the final pole of items for the national study. We had planned two approaches to select the best items. The first approach involved analysing the items in the context of respondents’ characteristics, such as gender, grade, self-reported difficulties in reading. The second approach involved statistical methods: factor analysis (FA) and Rasch analysis. Since it was preliminary analysis, also Item Response Theory (IRT) techniques (2PL model) were used to compare item characteristics under different assumptions. In the former method, we excluded items that did not load on the same factors as other items from the same set and did not align with the overall structure of the tool. Additionally, we analysed tetrachoric correlation coefficients between items and the percentage of correct responses.

### National study in schools

For the national survey, we selected 12- to 15-year-old pupils from sixth, seventh and eighth grades from 14 schools from large cities, 14 schools from medium-sized cities and 14 schools from rural communes located in 7 voivodeships chosen from all Polish macro-regions (Nomenclature of territorial units for statistics 1: major socio-economic regions, NUTS1) using the multistage random sampling technique. The data has been collected between May and June 2023 following the same procedure as in the pilot-testing survey by a team of trained interviewers according to a standardised protocol including collection of informed consent from parents and children. Besides the CET, the HLSAC-10 and 10 questions from the pilot survey, we added 2 questions regarding pupils’ ethnic background. 24 MCQs were presented to pupils in a random order.

This article has been developed and reported according to the COSMIN Reporting Guideline dedicated to studies on measurement properties of patient-reported outcome measures.^26^

### Methods of psychometric analysis

We tested the psychometric properties of the CET using, at first as a preliminary assessment, the Classical Test Theory and the Rasch analysis based on the assumption of adherence to the Guttman pattern, unidimensionality and local dependency.

### Classical Test Theory

The theoretical approach and the results of the previous research indicated unidimensionality of the analysed construct.^27^ Thus, we ran the confirmatory factor analysis (CFA) to verify the one-dimensional structure of the CET. We verified the goodness of fit of the proposed model to the data by the following indices: the root mean square error of approximation (RMSEA), the Comparative Fit Index (CFI) and Tucker-Lewis Index (TLI). The values of the RMSEA lower than 0.08, CFI and TLI higher than 0.90 indicated adequate fit.^28^ The analysis was done using the *lavaan* package in R software.

### Rasch analysis

We conducted the dichotomous Rasch model analysis using the TAM package.^29^ The model assumes that the probability that a person will affirm an item is a logistic function of the difference between the person’s level of CHL (person’s ability in this study) and the level of CHL expressed by the item.^30^

### Verification of the assumptions of unidimensionality and local dependency

Unidimensionality was verified in two ways. At first, as the proportion of variance in the responses associated with the Rasch model. The critical value of 20% of variance explained by the Rasch model is required as evidence of unidimensionality.^31^ Second, principal component analysis (PCA) of standardised residuals (difference between observed and expected values) was performed. This method verifies whether there is a pattern in the residuals, which might suggest the existence of an additional dimension, or whether there is rather random noise. The value of eigenvalues of the first contrast (first PCA of residuals component) should be lower than 1.5 to support the assumption of unidimensionality.^32^ Local dependency was tested using Yen’s Q3 based on the correlation of residuals. Residual correlations above 0.2 identify items that appear to be locally dependent.^33^

### Model-data fit

The model-data fit statistics included mean square error (MSE) and standardised versions of the Outfit and Infit statistics. These statistics provide a summary of the residual values associated with each item and person. The MSE versions of Outfit and Infit are expected to be close to 1.00, where the optimal value MSE versions of Outfit and Infit of each item should be located within 0.5–1.5.^34^ The values far greater than 1 indicate too much variation in the data and the values too close to 0 imply too high consistency. The standardised fit statistics are expected to be around 0.00 when data fit the Rasch model expectations. The marginal maximum likelihood estimation was applied.

### Reliability

The weighted likelihood estimate (WLE) person-separation and item-separation reliability and the Cronbach’s alpha coefficient were provided. Values above 0.7 are generally considered acceptable as evidence of reliability.

### Targeting

Targeting was evaluated using a visual distribution of both item difficulties and person abilities on the same logit scale. The item-person map (Wright map) was shown. It is expected that the mean of the logit scale locations of items and persons is around zero to indicate that the items are well-targeted, neither too easy nor too difficult for people in the sample.^30^

### Differential Item Functioning

DIF in relation to gender and grade was assessed. Calculations were done using the lordif package, which is a hybrid of logistic regression and item response theory.^35^ Three models were compared: (1) includes person ability only; (2) adds group membership to test for uniform DIF (item difficulty differs between groups in the same way across CHL level); (3) adds an interaction between ability and group to test for non-uniform DIF (the differences between groups vary across CHL level). A significant difference between Models 1 and 2 indicates uniform DIF, while a difference between Models 2 and 3 indicates non-uniform DIF. A McFadden pseudo R² value below 0.02 indicates negligible DIF, meaning there is little evidence that the item is interpreted differently across groups.^35^ Additionally, Monte Carlo simulations were used to obtain more accurate threshold values (online supplemental file 1).

### Convergent validity

As the HLSAC-10 assesses a similar construct and in the process of validation it exhibited sound psychometric properties in a similar population as targeted in this study,^25^ we used it to assess the construct of the CET. We assessed convergent validity by correlating the CET scores with the HLSAC-10 results using Pearson’s correlation coefficient. Additionally, we applied the Bland-Altman plot for assessing agreement between two measurement techniques.^36^ We expected the instruments to correlate positively; however, due to the different approaches towards health literacy (objective measurement in the CET vs subjective measurement in the HLSAC-10) and the characteristics of the HLSAC-10 presented in the literature,^24 25^ we assumed the correlation would be rather weak.^37 38^

### Known-group validity

We tested known-group validity by comparing the CET scores across gender and school grade groups. Based on other studies measuring health literacy, the following hypotheses were identified: (1) girls score higher on the CET than boys^18^; (2) respondents attending higher school grades score higher than respondents attending lower school grades.^25^ We used the t-test and analysis of variance to compare the CET score between the groups.

## Results

### Participants in the pilot and national studies

The pilot test was conducted on a sample of 303 pupils, of which 55.8% were girls and 1.3% chose not to inform about their gender. The mean age was 13.6 (median 13.4). Most of the included pupils attended sixth grade (44.2%), every fourth seventh grade (23.4%) and every third eighth grade (32.3%). More than half of the respondents went to school in middle-size cities (56.8%), every fourth in large cities (26.4%), every sixth in rural areas (16.8%). The average time to complete the test was 40 min (median 35 min), no missing data was found (table 2). The mean score of the CET with 36 MCQs was 18.9 (SD=5.1). Pupils’ impressions about the test shared with the moderators were mixed, ranging from ‘the test was long and difficult’ to ‘easy’ and ‘nice’. Observations from the pilot testing phase regarding the high percentage of pupils from refugees’ families resulted in adjusting the final questionnaire by adding two questions about the language spoken at home and the country of birth.

**Table 2:** The characteristics of participants and outcomes in the pilot and national studies

Characteristics		Pilot study N=303	National study N=2242
Gender N (%)	Boy	169 (55.8)	1055 (47.1)
	Girl	130 (42.9)	1055 (47.1)
	Not reported	4 (1.3)	132 (5.9)
Age (mean (SD))		13.6 (0.8)	12.8 (0.8)
School grade N (%)	Sixth	134 (44.2)	784 (35.0)
	Seventh	71 (23.4)	758 (33.8)
	Eighth	98 (32.3)	700 (31.2)
Place of residence N (%)	Rural	51 (16.8)	388 (17.3)
	Small and middle towns	172 (56.8)	1112 (49.6)
	Large town (>120 000 habitants)	80 (26.4)	742 (33.1)
HLSAC-10 mean (SD); min-max		28.8 (5.5); 10–40	28.8 (4.8); 10–40
CET mean (SD); min-max		13.8* (4.0); 5–22	13.0 (4.1); 1–23

* CET calculated as a sum of 24 items used in the national study.

CET, Claim Evaluation Tools; HLSAC, Health Literacy for School-Aged Children.

In the national study, 2242 pupils responded to the survey. 47.1% of the respondents were female, 5.9% chose not to provide information on their gender. The mean age was 12.8 (median 13.0). The percentage of respondents from sixth, seventh and eighth grades was similar, respectively 35.0%, 33.8%, 31.2%. No missing data was found (table 2).

Based on the analysis of 36 MCQs, we removed 12 MCQs (one per claim). Two items (Q1 and Q31) were removed due to very weak or reverse correlations with other items; six items (Q9, Q11, Q20, Q22, Q28, Q34) were removed due to low factor loadings in the performed FAs (based on the whole set, after removal of some items and in three-item sets); three items (Q4, Q14, Q26) were removed based on FA and IRT results; one item (Q17) was removed due to a high per cent of correct answers (90.5%).

### Validation

#### Classical Test Theory

We ran CFA to verify the unidimensionality of the tool tested on the national sample. The goodness of fit indices were: RMSEA=0.027, 90% CI=0.024 to 0.029; CFI=0.88; TLI=0.87; the Bayesian Information Criterion (BIC)=70 184.06. The fit of the model was improved after allowing error covariance between items Q9 and Q10: RMSEA=0.024, 90% CI=0.021 to 0.026; CFI=0.91; TLI=0.90; BIC=70 104.72, indicating that the one-factor model fitted the data.

14 out of 24 standardised factor loadings were greater than 0.3 (ranging from 0.313 to 0.490). The lowest factor loading was observed for Q7 (0.08), then for Q5 (0.19). The items: Q8-Q10, Q13, Q17, Q18, Q22, Q23 had factor loadings between 0.2 and 0.3. The MCQ wording is provided in table 4.

#### Rasch analysis

The analysis showed that 20% of the variance in pupils’ responses can be explained by the Rasch model estimates, which is a minimum to support the evidence of unidimensionality. All of the contrasts have an eigenvalue lower than 1.5 (the first eigenvalue=1.4, the second eigenvalue=1.3), which met the criteria for unidimensionality. The Yen’s Q3 values range from −0.10 to −0.17 (mean=−0.03, SD=0.04), thus the assumption of local independence was met.

#### Model-data fit

The results showed that the most difficult item is Q18 (proportion correct=18%; δ=1.66; SE=0.06) followed by Q7 (proportion correct=32%; δ=0.82; SE=0.05), while the easiest item is Q14 (proportion correct*=*73%; δ=−1.12; SE=0.05) followed by Q10 (proportion correct=73%; δ=−1.10; SE=0.05) (table 4). The average Infit and Outfit mean square statistics were around 1.00, and average standardised Infit and Outfit statistics were near the expected value of 0.00 when data fit the model, which implied adequate model fit (table 3). The MSE Outfit statistics were ranging from 0.89 (Q15) to 1.16 (Q7). The MSE Infit statistics ranged from 0.92 (Q15) to 1.11 (Q7), which indicated that all items were inside the suggested range (table 4).

**Table 3:** Model summary table

Statistic	Items	Persons
Logit scale location mean	−0.29	0.00
Logit scale location SD	0.66	0.85
SEM	0.05	0.46
SE SD	0.00	0.07
Outfit MSE mean	1.00	0.97
Outfit MSE SD	0.06	0.20
Infit MSE mean	1.00	0.98
Infit MSE SD	0.04	0.14
Standard Outfit mean	−0.01	−0.07
Standard Outfit SD	3.66	0.85
Standard Infit mean	0.04	−0.06
Standard Infit SD	2.53	0.82
Reliability of separation	0.71	0.70

MSE, mean square error.

**Table 4:** Summary table of item-model fit statistics

Multiple-choice questions	Proportion correct	Item location (*δ*)	Item SE	Outfit MSE	Standard Outfit	Infit MSE	Standard Infit
Q18. Dissimilar comparison groups	0.18	1.66	0.06	0.98	−0.58	0.98	−0.55
Q7. Old is better!	0.32	0.82	0.05	1.16	7.88	1.11	5.46
Q8. Old is better!	0.37	0.58	0.05	1.09	5.04	1.04	2.6
Q17. Dissimilar comparison groups	0.41	0.41	0.05	1.06	4.25	1.04	2.48
Q4. No comparison needed!	0.48	0.07	0.04	0.98	−1.55	0.98	−1.45
Q23. Do the advantages outweigh the disadvantages?	0.5	0.01	0.04	1.05	3.81	1.04	3.07
Q9. New is better!	0.53	−0.12	0.04	1.02	1.70	1.02	1.43
Q24. Do the advantages outweigh the disadvantages?	0.54	−0.17	0.04	0.99	−0.91	0.99	−0.54
Q12. As advertised!	0.54	−0.19	0.04	1.01	0.71	1.01	0.89
Q13. It worked for me!	0.55	−0.22	0.04	1.05	3.31	1.04	2.61
Q5. A study shows!	0.56	−0.29	0.04	1.08	5.83	1.06	4.29
Q21. Few people or events	0.57	−0.32	0.05	0.97	−2.36	0.98	−1.56
Q6. A study shows!	0.60	−0.44	0.05	0.98	−1.27	0.98	−1.11
Q3. No comparison needed!	0.60	−0.46	0.05	0.98	−1.21	0.99	−0.59
Q16. Recommended by expert!	0.60	−0.48	0.05	0.91	−5.84	0.93	−4.4
Q19. Dissimilar expectations	0.62	−0.55	0.05	0.94	−3.65	0.96	−2.59
Q22. Few people or events	0.65	−0.71	0.05	1.03	1.93	1.03	1.68
Q15. Recommended by expert!	0.66	−0.72	0.05	0.89	−6.53	0.92	−4.71
Q2 100% safe!	0.67	−0.80	0.05	0.99	−0.31	0.99	−0.35
Q1 100% safe!	0.69	−0.89	0.05	0.97	−1.64	0.98	−1.22
Q20. Dissimilar expectations	0.70	−0.96	0.05	0.98	−0.83	0.99	−0.55
Q11. As advertised!	0.72	−1.07	0.05	0.93	−3.15	0.96	−1.63
Q10. New is better!	0.73	−1.10	0.05	0.99	−0.24	1.00	−0.14
Q14. It worked for me!	0.73	−1.12	0.05	0.90	−4.57	0.95	−2.21

MSE, mean square error.

#### Reliability

WLE reliability coefficients were 0.70 and 0.71 (table 3), the Cronbach’s alpha was 0.72 which suggests good reliability of the scale.

### Targeting

**Figure 2:**
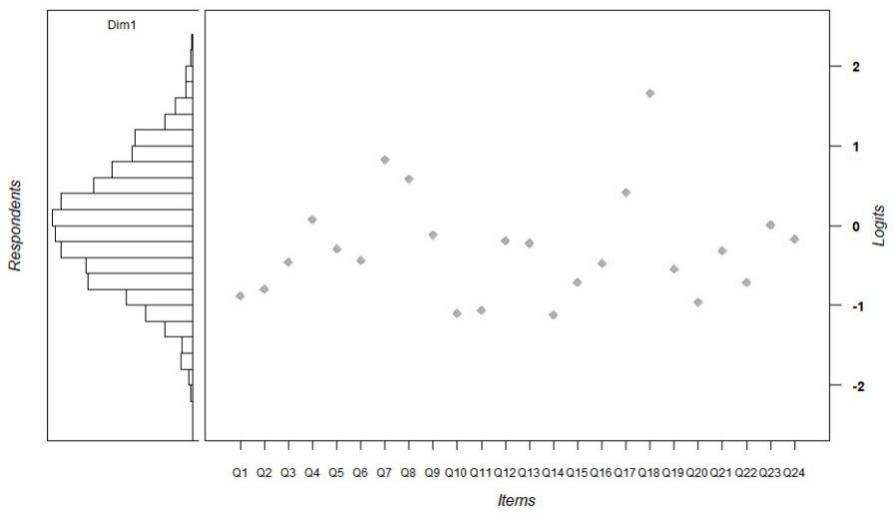
The item-person map for the Claim Evaluation Tools 24-item scale.

Figure 2 presents visualisation and comparison of the item and person location on the single linear continuum. The left panel of **figure 2** shows a histogram of respondent (person) locations on the logit scale that represents the latent variable (critical health literacy measured by the CET). The large central panel of the plot shows the item locations (item difficulty estimates) on the logit scale that represents the level of CHL. On average, pupils were located slightly higher on the logit scale (M=0.00, SD=0.85) than the items (M=−0.29, SD=0.66), but the difference between means of location parameters is relatively small. It suggests that the chosen items were well-targeted, not too easy and not too difficult for the pupils under study. Average values of the SE were slightly larger for pupils (M=0.46) compared with items (M=0.05), indicating that there may be some issues related to targeting for some of the pupils who participated in the assessment (table 3).

### Differential Item Functioning

Statistically significant DIF by gender was shown in relation to items: Q23, Q10, Q6, Q2 (uniform) and Q1, Q9, Q18 (non-uniform). Nonetheless, all pseudo R^2^ statistics are lower than 0.01. Further Monte Carlo simulations showed that some pseudo R^2^ values varied across items, but overall, they were very small under simulations that assume no DIF. For instance, the maximum pseudo R^2^ in online supplemental table S1 (see supplement 1) was 0.007, and thus a reasonable lower bound that would avoid type I errors might be 0.01. In regard to grade, statistically significant DIF was shown in relation to items: Q17, Q18, Q22 (uniform) and Q14 (uniform and non-uniform) (online supplemental file 1). Importantly, none of the pseudo R² statistics exceed 0.008. Further Monte Carlo simulations showed that some pseudo R^2^ values varied across items, but overall, they were also very small under simulations that assume no DIF. The maximum pseudo R^2^ was 0.009, which interestingly corresponds to a small, non-negligible effect size.^39^ Thus, the results indicate a lack of evidence of differential interpretation of the CET scale items across the tested the tested gender and grade groups.

### Scoring rule

Since the final set of 24 items fits to the Rasch model, the final CET score as a summary of correct answers is justified. The scoring rule is to sum up the correct responses from the items.

### Convergent validity

Pearson’s correlation coefficient equals 0.223, which indicates a rather poor linear association between these two scales.^40^ We transformed the scores of the CET and the HLSAC-10 scale to 0–100. The mean difference between the CET (mean=54.7) and the HLSAC-10 (mean=62.6) is −7.9. The results suggest that pupils tended to report higher self-assessed health literacy scores on the HLSAC-10 compared with their objective CET scores. There is a systematic difference. There are many points which are not in between the upper and lower 95% limit of agreement (online supplemental file 2).

### Known-group validity

The analysis of the known group differences confirmed the significant differences in gender and school grade groups. Girls scored significantly higher than boys (p<0.001), and pupils attending higher grades scored significantly higher than those attending the lower grades (p<0.001) (table 5).

**Table 5:** The differences in Claim Evaluation Tools between gender (t-test) and grade groups (analysis of variance)

Variables		N (%)	Summary score	P value
			Mean (SD)	
Gender	Boys	1055 (47.1)	12.51 (3.94)	<0.001
	Girls	1055 (47.1)	13.66 (3.94)	
School grade	Sixth	784 (35.0)	12.66 (3.94)	<0.001
	Seventh	758 (33.8)	13.11 (4.10)	
	Eighth	700 (31.2)	13.40 (4.14)	

## Discussion

We translated and adapted the first objective measurement tool for evaluating CHL to the Polish cultural context. Using data from a national sample of primary school pupils we assessed the psychometric properties of the Polish version of the CET. We confirmed the unidimensionality of the tool and assessed the model fit as satisfying.

Our analysis suggested that the tested MCQs were neither too simple nor too challenging for the sample studied. The most difficult (answered correctly by less than 50% of the respondents in the national sample), turned out to be two MCQs referring to the concept about evidence indicating that comparison groups should be as similar as possible (Q18, Q17) and two MCQs reflecting the claim ‘Old is better!’ (Q7, Q8). Additionally, less than half of the pupils chose the correct answer in MCQ illustrating the claim ‘No comparison needed’ (Q4). The MCQs correctly responded by the majority of the pupils (more than 70% of respondents) recognised the unreliable claims about the effects of interventions: ‘As advertised!’ (Q11), ‘New is better!’ (Q10) and ‘It worked for me!’ (Q14). Among the most difficult MCQs, two items linked to the similarity of the comparison group and the crucial role of making comparisons (Q18, Q17) were significantly easier for boys than girls, and among the easiest MCQs, the claim ‘New is better!’ (Q10) was significantly easier for girls.

While the difficulty in understanding the importance of making comparisons might result from the lack of scientific education in the primary school curricula in Poland,^41^ the slight differences between the level of difficulty for boys and girls might reflect the dissimilar socialisation experiences, including developmental stimuli available for girls and boys, after school curricula or dominant interests in their peer groups. We found that several items exhibited DIFs, but the differences in the item parameters between genders and grade were not considered as meaningful. We considered the question of whether the particular items should be removed from the scale due to the culturally determined bias linked to gender differences or age differences, or whether there is a factual difference and removing the item would render the scale incomplete.

The psychometric properties of the Polish version of the CET are satisfying. The reliability is good and its value corresponds with the results from other countries.^16 19^ Consistent with previous studies, girls and older respondents performed better in the CET than boys and younger pupils, respectively.^18^ Finally, the weak correlation between the CET and the HLSAC-10 confirmed some level of convergent validity. The weakness of the relationship might have resulted from the different conceptualisations and operationalisations of health literacy applied in those tools. While both of the tools assess young people’s health literacy, the CET focuses on one dimension of CHL and the HLSAC studies HL more broadly by looking into five diverse dimensions. The CET objectively measures the actual knowledge and ability to recognise the health claims, while in the HLSAC-10 people self-assess their abilities linked to the health domain.^10^

This study has several strengths. To ensure a comprehensive approach to cultural adaptation, the processes involved the interdisciplinary team of experts and researchers. The clarity of the translated items was verified based on experts’ and pupils’ feedback. For the sake of methodological rigour, the psychometric properties of the CET were analysed based on a representative sample of adolescents. One limitation of the study is the lack of verification of respondents’ health status, which made it impossible to plan procedures for recruiting pupils participating in individualised education programmes who, due to health-related conditions, could not be present in the school classrooms during data collection. As a result, subgroup analysis between adolescents with and without health problems could not be presented. Further studies should examine the learning and memory effect on the CET outcomes as well as explore the needs of pupils with special needs regarding their ability to navigate the CET.

### Conclusions

The findings of this study support the validity and reliability of the adapted version of the CET. The Polish version of the CET is appropriate to use in the population of 12- to 15-year-old adolescents to measure CHL. The tool can be used in educational and research settings.

## Supplementary material

10.1136/bmjopen-2025-099994 online supplemental file 1

10.1136/bmjopen-2025-099994 online supplemental file 2

## Data Availability

All data relevant to the study are included in the article or uploaded as supplementary information.
