# Supplementary material for: Measurement of critical health literacy in primary school pupils: a Polish validation of the Claim Evaluation Tools
Source: BMJ Open. 2025 Jul 17;15(7):e099994. doi: 10.1136/bmjopen-2025-099994 (PMC12273065; doi:10.1136/bmjopen-2025-099994)

Supplement 1

Differential item functioning in relation to gender and grade.

Calculations were done using *lordif* package, which is a hybrid of logistic regression and item response theory.

Four models are considered:

*“Model 0 : logit P(ui ≥ k) = _k*

*Model 1 : logit P(ui ≥ k) = _k + β1 *ability*

*Model 2 : logit P(ui ≥k) = _k + β1 * ability + β2 * group*

*Model 3 : logit P(ui ≥k) = _k + β1 * ability + β2 * group + β2 * ability * group;*

*where P(ui ≥ k) denotes the cumulative probabilities that the actual item response ui falls in*

*category k or higher.”*

Significant difference between model 2 and model 1 implicates uniform DIF. Significant difference between model 3 and model 2 implicates nonuniform DIF. A value of McFadden pseudo *R*2 (< 0.02) indicates a lack of evidence of differential interpretation of an item across the tested groups.

## Differential item functioning in relation to gender


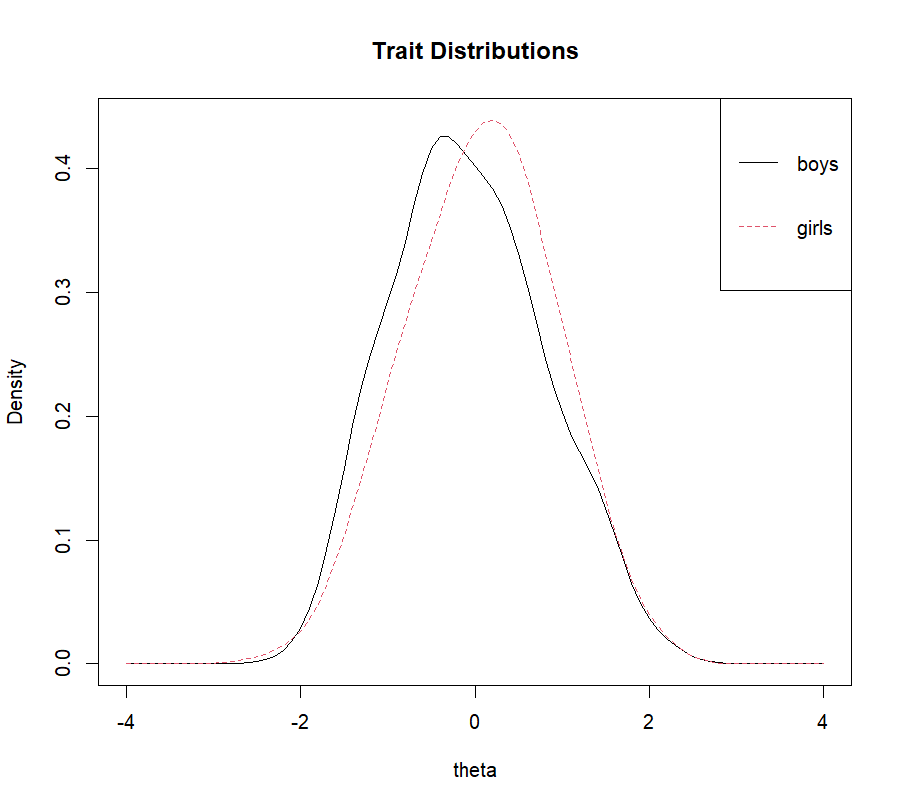


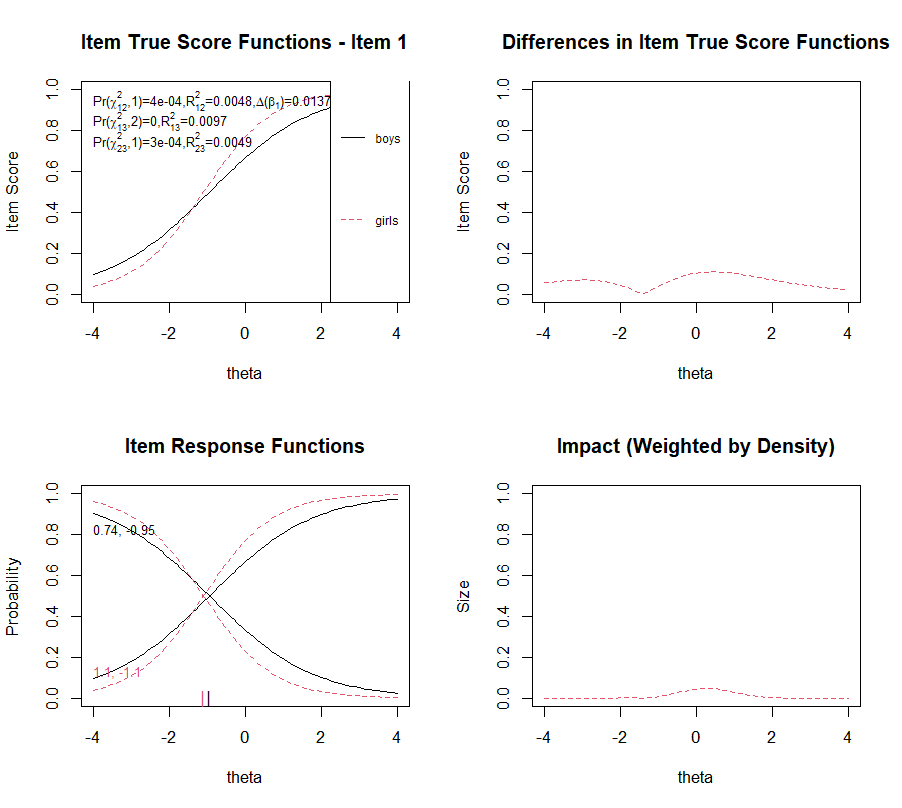


Differences between model 2 and 3 is significant, which suggests non-uniform dif. The differences between sloes are not very large (0.74 vs. 1.1) as well as between thresholds ( -0.95 vs. -1.1). There is a difference between the item- true score functions peaking at approximately theta= 0.5, whereas the density weighted impact seems to be negligible. It is also observed small McFadden's pseudo R2 measures (printed on the top left plot), i.e., R^2^13 = 0:010 and R^2^23 = 0:005.

The results are presented only for the items which show statistically significant DIFs.


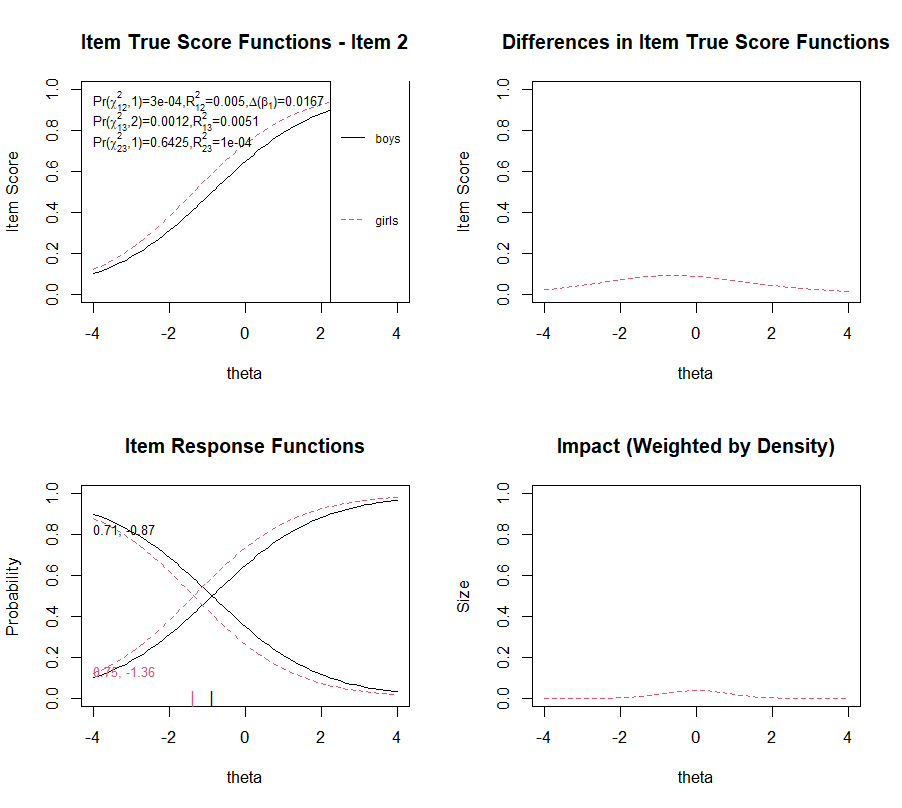
 McFadden's R2 change for uniform DIF was 0:005, which is considered a negligible effect size (Cohen 1988).


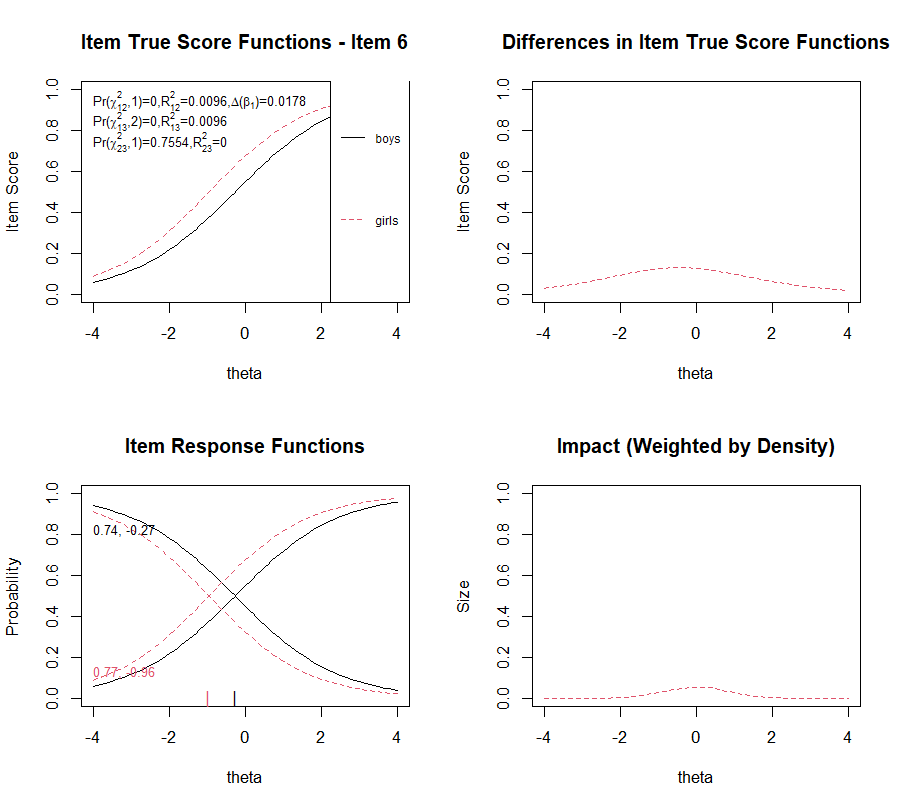

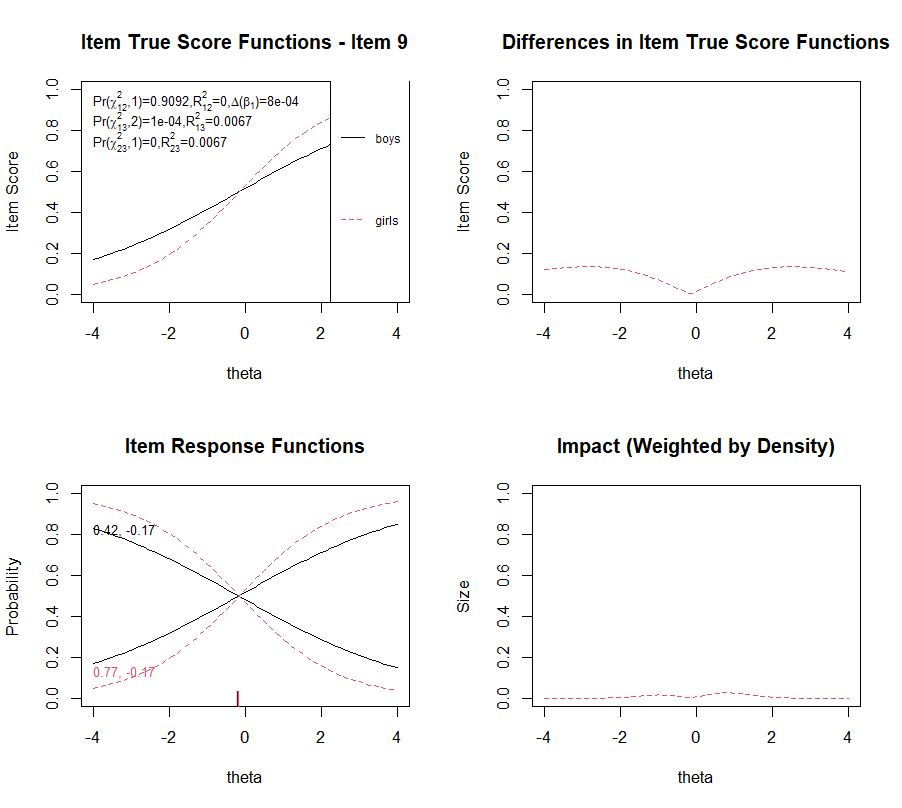

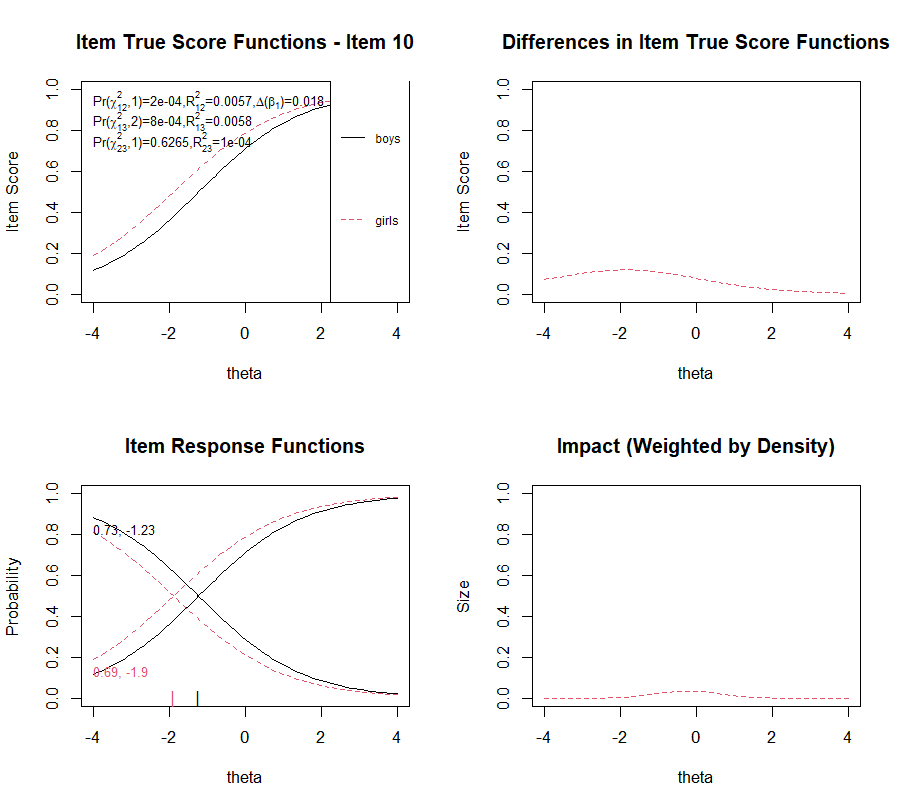

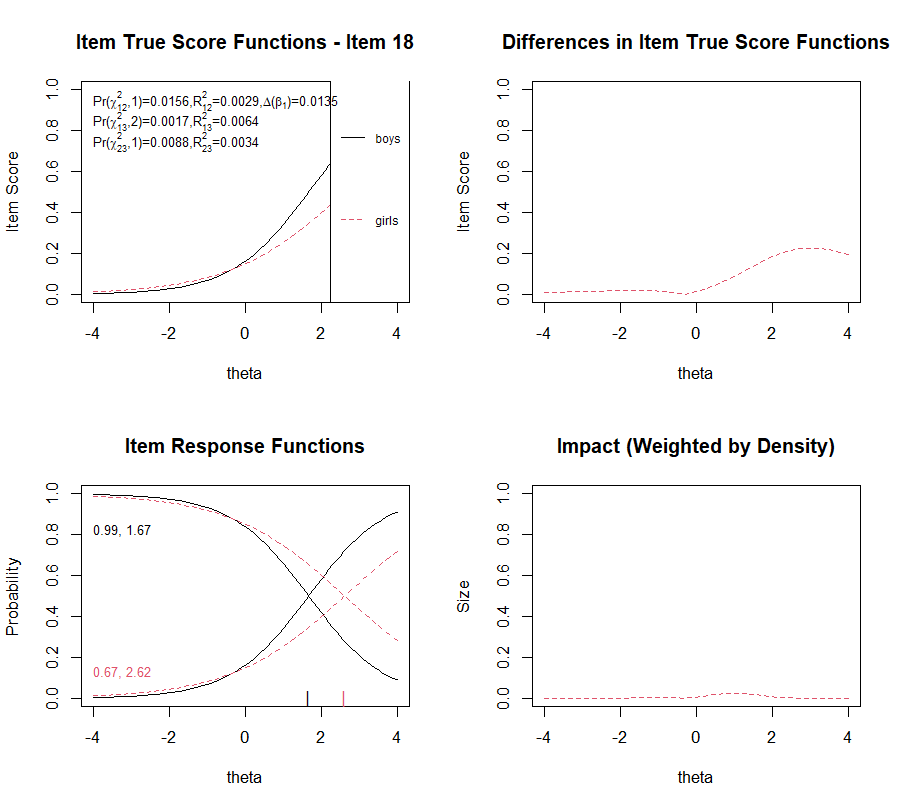

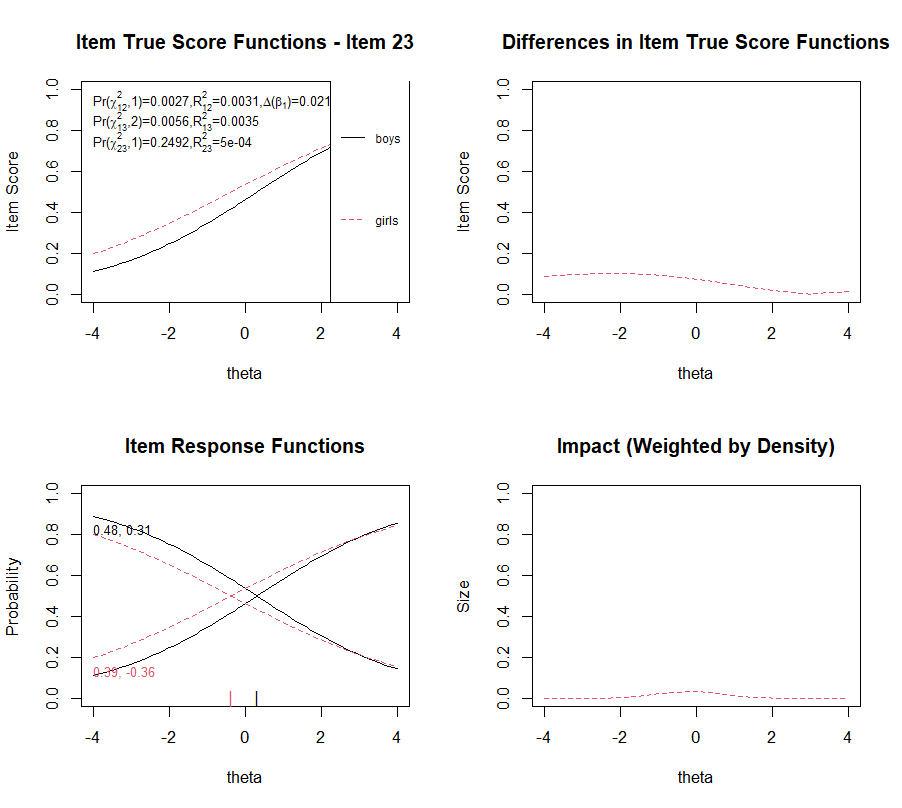


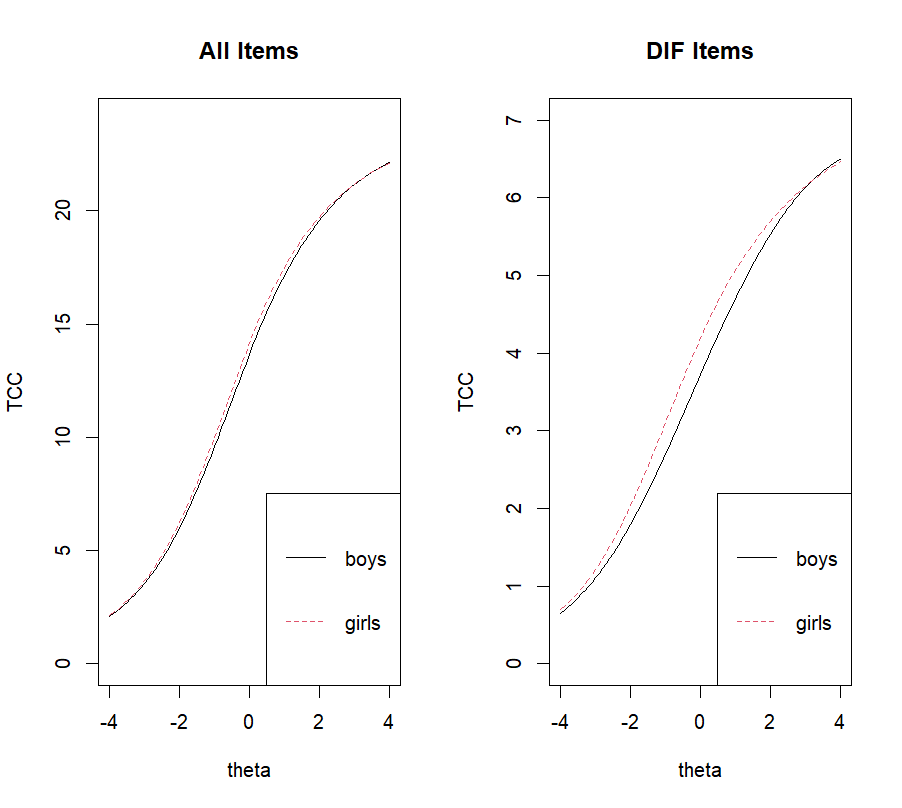


| Table S1. Empirical threshold values for DIF detection by gender based on Monte Carlo simulations (nr = 1000, alpha =0.01). | | | | | | | | | | | | | |
| --- | --- | --- | --- | --- | --- | --- | --- | --- | --- | --- | --- | --- | --- |
|  | chi12 | chi13 | chi23 | pseudo12.CoxSnell | pseudo13.CoxSnell | pseudo23.CoxSnell | pseudo12.Nagelkerke | pseudo13.Nagelkerke | pseudo23.Nagelkerke | pseudo12.McFadden | pseudo13.McFadden | pseudo23.McFadden | beta12 |
| Q1 | 0.0126 | 0.0151 | 0.0097 | 0.0026 | 0.0034 | 0.0027 | 0.0037 | 0.0048 | 0.0038 | 0.0024 | 0.0032 | 0.0026 | 0.0149 |
| Q2 | 0.0127 | 0.0140 | 0.0116 | 0.0026 | 0.0036 | 0.0026 | 0.0036 | 0.0051 | 0.0036 | 0.0024 | 0.0032 | 0.0024 | 0.0160 |
| Q3 | 0.0138 | 0.0160 | 0.0159 | 0.0025 | 0.0034 | 0.0024 | 0.0034 | 0.0046 | 0.0032 | 0.0021 | 0.0029 | 0.0021 | 0.0155 |
| Q4 | 0.0047 | 0.0088 | 0.0094 | 0.0032 | 0.0038 | 0.0026 | 0.0043 | 0.0051 | 0.0035 | 0.0027 | 0.0032 | 0.0023 | 0.0136 |
| Q5 | 0.0120 | 0.0129 | 0.0089 | 0.0029 | 0.0040 | 0.0031 | 0.0039 | 0.0053 | 0.0042 | 0.0022 | 0.0030 | 0.0024 | 0.0269 |
| Q6 | 0.0069 | 0.0101 | 0.0121 | 0.0030 | 0.0038 | 0.0026 | 0.0040 | 0.0051 | 0.0035 | 0.0026 | 0.0032 | 0.0022 | 0.0149 |
| Q7 | 0.0114 | 0.0108 | 0.0116 | 0.0030 | 0.0042 | 0.0030 | 0.0043 | 0.0060 | 0.0041 | 0.0025 | 0.0034 | 0.0024 | 0.0624 |
| Q8 | 0.0094 | 0.0115 | 0.0164 | 0.0031 | 0.0040 | 0.0026 | 0.0042 | 0.0055 | 0.0035 | 0.0024 | 0.0032 | 0.0021 | 0.0228 |
| Q9 | 0.0101 | 0.0158 | 0.0093 | 0.0028 | 0.0036 | 0.0030 | 0.0038 | 0.0049 | 0.0039 | 0.0023 | 0.0028 | 0.0023 | 0.0180 |
| Q10 | 0.0103 | 0.0077 | 0.0119 | 0.0028 | 0.0041 | 0.0027 | 0.0041 | 0.0060 | 0.0040 | 0.0027 | 0.0040 | 0.0026 | 0.0166 |
| Q11 | 0.0067 | 0.0069 | 0.0148 | 0.0029 | 0.0039 | 0.0024 | 0.0042 | 0.0058 | 0.0035 | 0.0029 | 0.0042 | 0.0025 | 0.0158 |
| Q12 | 0.0108 | 0.0107 | 0.0102 | 0.0027 | 0.0038 | 0.0028 | 0.0036 | 0.0051 | 0.0037 | 0.0022 | 0.0031 | 0.0023 | 0.0158 |
| Q13 | 0.0124 | 0.0129 | 0.0104 | 0.0028 | 0.0038 | 0.0029 | 0.0037 | 0.0051 | 0.0039 | 0.0021 | 0.0030 | 0.0023 | 0.0199 |
| Q14 | 0.0122 | 0.0124 | 0.0122 | 0.0024 | 0.0033 | 0.0024 | 0.0035 | 0.0048 | 0.0035 | 0.0026 | 0.0036 | 0.0026 | 0.0132 |
| Q15 | 0.0102 | 0.0113 | 0.0127 | 0.0023 | 0.0031 | 0.0021 | 0.0032 | 0.0042 | 0.0030 | 0.0025 | 0.0034 | 0.0023 | 0.0115 |
| Q16 | 0.0082 | 0.0062 | 0.0078 | 0.0025 | 0.0036 | 0.0024 | 0.0033 | 0.0049 | 0.0033 | 0.0025 | 0.0036 | 0.0025 | 0.0119 |
| Q17 | 0.0094 | 0.0112 | 0.0097 | 0.0030 | 0.0040 | 0.0029 | 0.0040 | 0.0053 | 0.0040 | 0.0024 | 0.0032 | 0.0024 | 0.0202 |
| Q18 | 0.0107 | 0.0065 | 0.0057 | 0.0028 | 0.0043 | 0.0033 | 0.0047 | 0.0073 | 0.0054 | 0.0034 | 0.0052 | 0.0039 | 0.0155 |
| Q19 | 0.0106 | 0.0153 | 0.0117 | 0.0026 | 0.0033 | 0.0024 | 0.0035 | 0.0045 | 0.0033 | 0.0024 | 0.0030 | 0.0023 | 0.0127 |
| Q20 | 0.0067 | 0.0058 | 0.0065 | 0.0030 | 0.0043 | 0.0030 | 0.0043 | 0.0062 | 0.0043 | 0.0029 | 0.0040 | 0.0029 | 0.0186 |
| Q21 | 0.0158 | 0.0085 | 0.0107 | 0.0023 | 0.0036 | 0.0026 | 0.0031 | 0.0049 | 0.0035 | 0.0020 | 0.0033 | 0.0023 | 0.0121 |
| Q22 | 0.0098 | 0.0134 | 0.0103 | 0.0030 | 0.0038 | 0.0029 | 0.0041 | 0.0053 | 0.0040 | 0.0024 | 0.0032 | 0.0024 | 0.0215 |
| Q23 | 0.0081 | 0.0070 | 0.0096 | 0.0031 | 0.0045 | 0.0030 | 0.0042 | 0.0060 | 0.0040 | 0.0024 | 0.0034 | 0.0023 | 0.0215 |
| Q24 | 0.0069 | 0.0097 | 0.0089 | 0.0031 | 0.0038 | 0.0029 | 0.0041 | 0.0051 | 0.0039 | 0.0025 | 0.0032 | 0.0024 | 0.0167 |
| mean | 0.0101 | 0.0109 | 0.0108 | 0.0028 | 0.0038 | 0.0027 | 0.0039 | 0.0053 | 0.0038 | 0.0025 | 0.0034 | 0.0025 | 0.0187 |
| sd | 0.0026 | 0.0032 | 0.0026 | 0.0003 | 0.0004 | 0.0003 | 0.0004 | 0.0007 | 0.0005 | 0.0003 | 0.0005 | 0.0004 | 0.0101 |

### Summary:

Based on the analysis statistically significant differences item functioning were shown in relation to questions: 23, 10, 6, 2 (uniform) and 1,9,18 (nonuniform). Nonetheless, all pseudo R2 statistics are lower than 0.01.

Further Monte Carlo simulations showed that some pseudo R2 values varied across questions, but overall, they were very small under simulations that assume no DIF. For instance, the maximum pseudo R2 in Table 1 was 0.007, and thus a reasonable lower bound that would avoid Type I errors might be 0.01, which is lower than 0.2, which interestingly corresponds to a small, non-negligible effect size (Cohen 1988).

Thus, the results indicates a lack of evidence of differential interpretation of an item across the tested groups.

## Differential item functioning in relation to grade


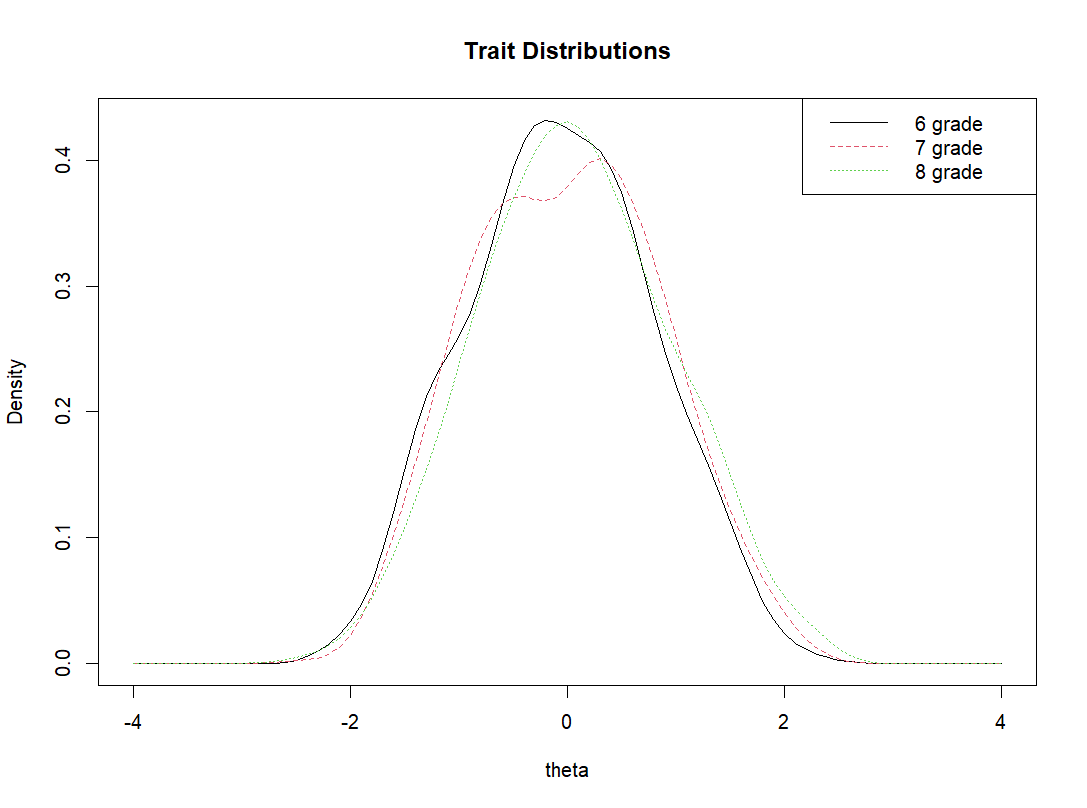


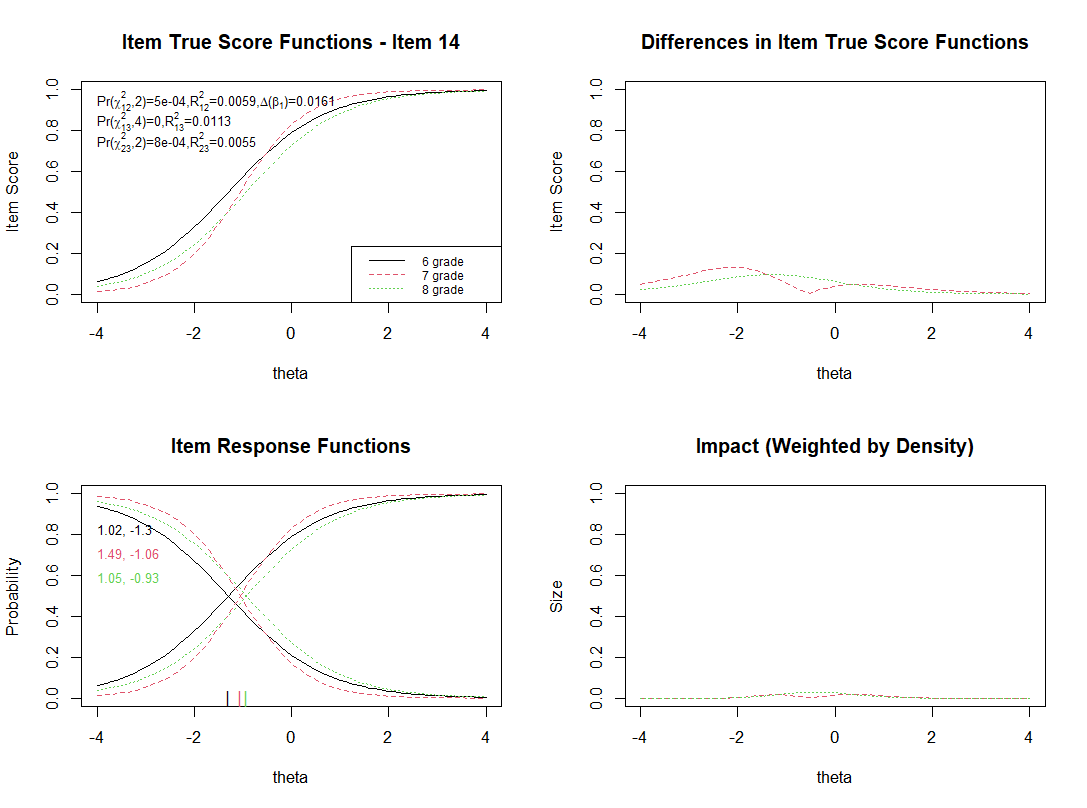


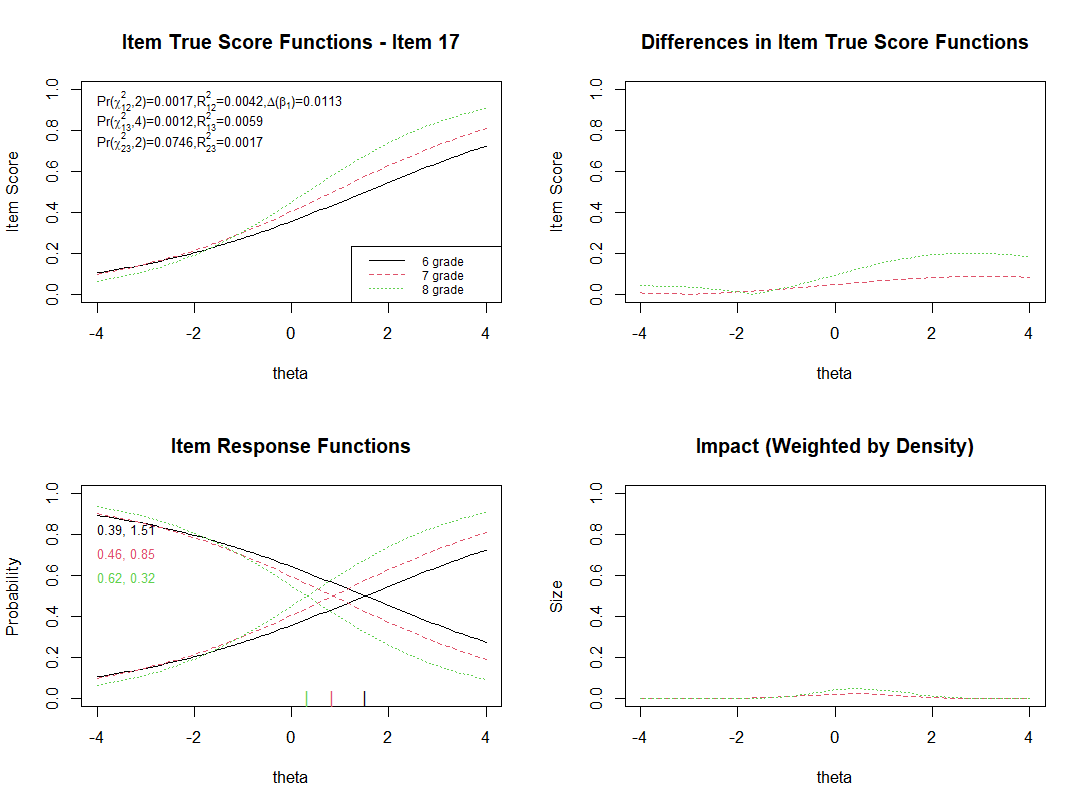


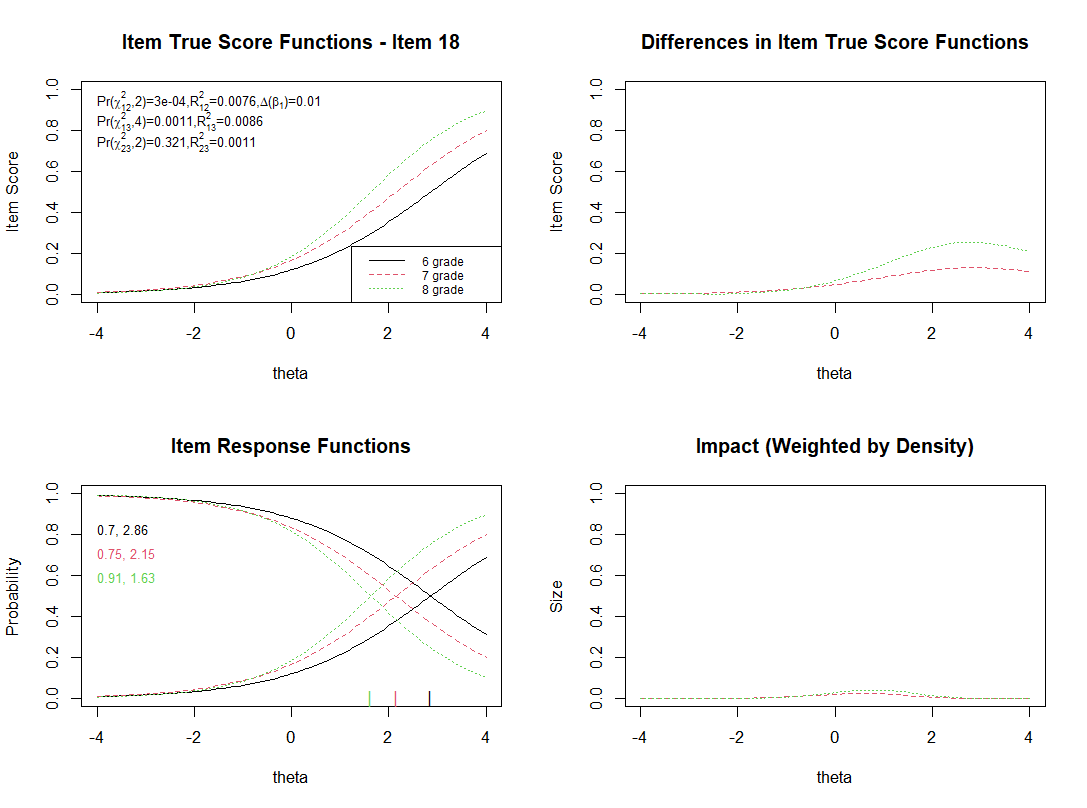


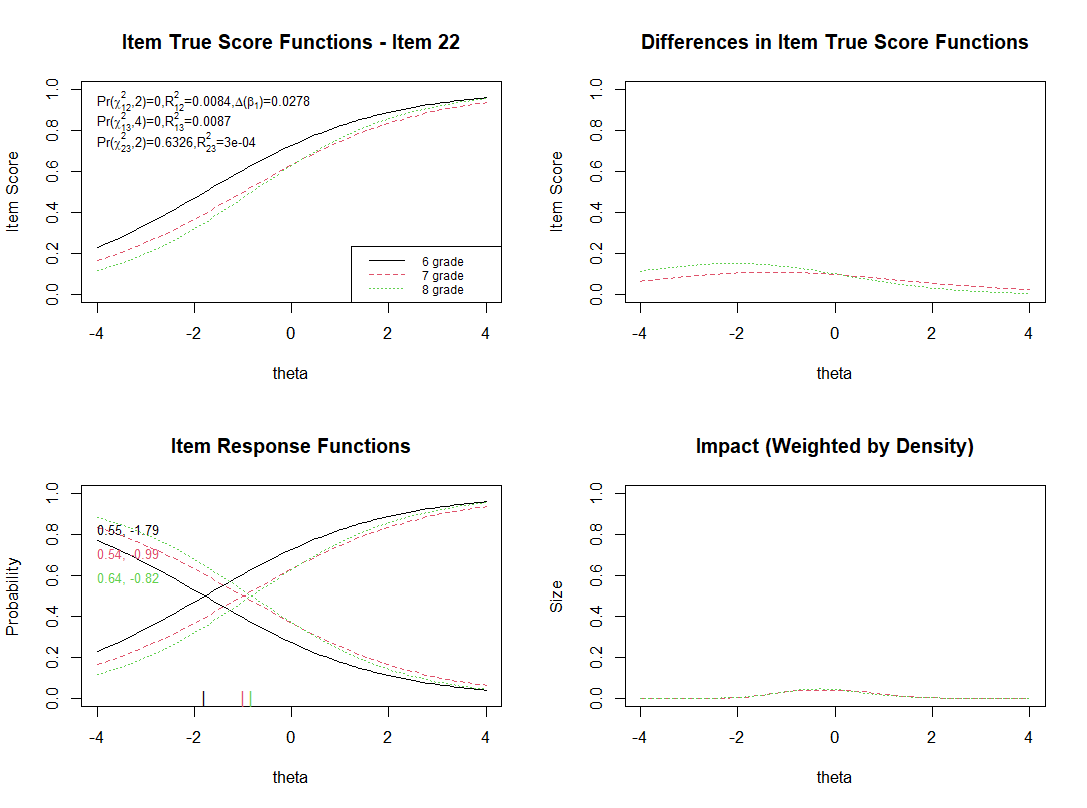


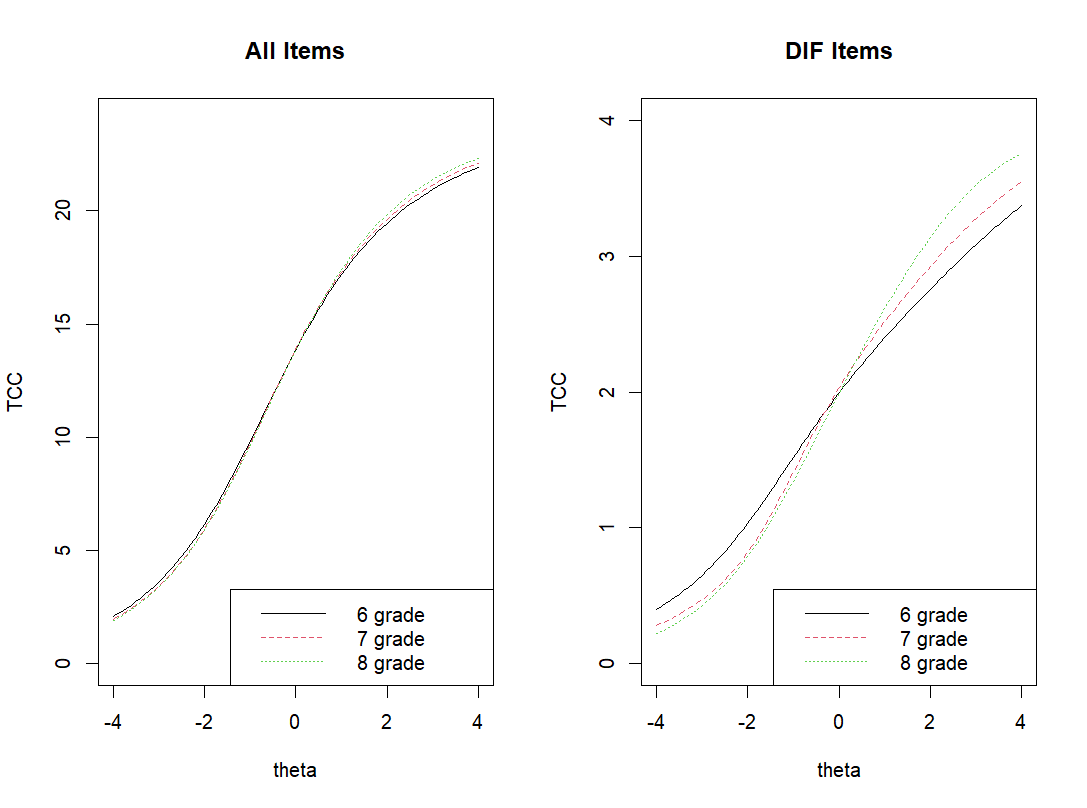


Supplementary Table 2. Empirical threshold values for DIF detection by grade based on Monte Carlo simulations (nr = 1000, alpha =0.01).

|  | chi12 | chi13 | chi23 | pseudo12.CoxSnell | pseudo13.CoxSnell | pseudo23.CoxSnell | pseudo12.Nagelkerke | pseudo13.Nagelkerke | pseudo23.Nagelkerke | pseudo12.McFadden | pseudo13.McFadden | pseudo23.McFadden | beta12 |
| --- | --- | --- | --- | --- | --- | --- | --- | --- | --- | --- | --- | --- | --- |
| Q1 | 0.0128 | 0.0118 | 0.0130 | 0.0033 | 0.0048 | 0.0032 | 0.0047 | 0.0069 | 0.0046 | 0.0032 | 0.0047 | 0.0031 | 0.0090 |
| Q2 | 0.0092 | 0.0079 | 0.0146 | 0.0037 | 0.0055 | 0.0033 | 0.0052 | 0.0076 | 0.0046 | 0.0033 | 0.0049 | 0.0030 | 0.0111 |
| Q3 | 0.0107 | 0.0105 | 0.0080 | 0.0036 | 0.0051 | 0.0037 | 0.0048 | 0.0069 | 0.0051 | 0.0030 | 0.0044 | 0.0032 | 0.0098 |
| Q4 | 0.0098 | 0.0101 | 0.0064 | 0.0035 | 0.0049 | 0.0038 | 0.0047 | 0.0066 | 0.0050 | 0.0030 | 0.0043 | 0.0033 | 0.0095 |
| Q5 | 0.0131 | 0.0097 | 0.0050 | 0.0037 | 0.0057 | 0.0046 | 0.0050 | 0.0077 | 0.0061 | 0.0028 | 0.0044 | 0.0035 | 0.0159 |
| Q6 | 0.0106 | 0.0094 | 0.0106 | 0.0035 | 0.0051 | 0.0035 | 0.0047 | 0.0068 | 0.0047 | 0.0030 | 0.0044 | 0.0030 | 0.0084 |
| Q7 | 0.0087 | 0.0104 | 0.0072 | 0.0042 | 0.0058 | 0.0044 | 0.0059 | 0.0082 | 0.0060 | 0.0034 | 0.0048 | 0.0034 | 0.0452 |
| Q8 | 0.0100 | 0.0082 | 0.0135 | 0.0039 | 0.0058 | 0.0037 | 0.0053 | 0.0079 | 0.0050 | 0.0031 | 0.0047 | 0.0029 | 0.0148 |
| Q9 | 0.0079 | 0.0159 | 0.0113 | 0.0039 | 0.0051 | 0.0036 | 0.0053 | 0.0068 | 0.0048 | 0.0031 | 0.0039 | 0.0029 | 0.0123 |
| Q10 | 0.0101 | 0.0140 | 0.0115 | 0.0037 | 0.0050 | 0.0035 | 0.0054 | 0.0073 | 0.0051 | 0.0036 | 0.0048 | 0.0034 | 0.0120 |
| Q11 | 0.0149 | 0.0114 | 0.0156 | 0.0031 | 0.0049 | 0.0031 | 0.0046 | 0.0072 | 0.0046 | 0.0032 | 0.0050 | 0.0032 | 0.0090 |
| Q12 | 0.0118 | 0.0127 | 0.0111 | 0.0035 | 0.0050 | 0.0036 | 0.0047 | 0.0067 | 0.0048 | 0.0029 | 0.0041 | 0.0029 | 0.0106 |
| Q13 | 0.0128 | 0.0186 | 0.0183 | 0.0037 | 0.0050 | 0.0033 | 0.0049 | 0.0067 | 0.0044 | 0.0028 | 0.0039 | 0.0026 | 0.0125 |
| Q14 | 0.0111 | 0.0134 | 0.0169 | 0.0032 | 0.0045 | 0.0030 | 0.0047 | 0.0067 | 0.0044 | 0.0035 | 0.0050 | 0.0032 | 0.0092 |
| Q15 | 0.0054 | 0.0106 | 0.0139 | 0.0034 | 0.0043 | 0.0027 | 0.0047 | 0.0059 | 0.0038 | 0.0036 | 0.0046 | 0.0030 | 0.0084 |
| Q16 | 0.0139 | 0.0160 | 0.0131 | 0.0028 | 0.0041 | 0.0028 | 0.0038 | 0.0055 | 0.0039 | 0.0029 | 0.0041 | 0.0029 | 0.0075 |
| Q17 | 0.0111 | 0.0111 | 0.0159 | 0.0038 | 0.0054 | 0.0034 | 0.0051 | 0.0073 | 0.0046 | 0.0030 | 0.0043 | 0.0027 | 0.0151 |
| Q18 | 0.0099 | 0.0119 | 0.0165 | 0.0037 | 0.0052 | 0.0033 | 0.0061 | 0.0086 | 0.0056 | 0.0044 | 0.0062 | 0.0041 | 0.0129 |
| Q19 | 0.0075 | 0.0119 | 0.0198 | 0.0036 | 0.0047 | 0.0028 | 0.0049 | 0.0065 | 0.0039 | 0.0033 | 0.0044 | 0.0026 | 0.0090 |
| Q20 | 0.0104 | 0.0127 | 0.0129 | 0.0036 | 0.0050 | 0.0034 | 0.0053 | 0.0071 | 0.0048 | 0.0035 | 0.0047 | 0.0032 | 0.0110 |
| Q21 | 0.0091 | 0.0097 | 0.0100 | 0.0035 | 0.0049 | 0.0035 | 0.0047 | 0.0066 | 0.0046 | 0.0031 | 0.0043 | 0.0030 | 0.0088 |
| Q22 | 0.0115 | 0.0094 | 0.0076 | 0.0037 | 0.0055 | 0.0040 | 0.0051 | 0.0077 | 0.0055 | 0.0031 | 0.0047 | 0.0034 | 0.0123 |
| Q23 | 0.0074 | 0.0073 | 0.0142 | 0.0041 | 0.0059 | 0.0036 | 0.0054 | 0.0078 | 0.0048 | 0.0032 | 0.0045 | 0.0027 | 0.0154 |
| Q24 | 0.0092 | 0.0077 | 0.0086 | 0.0037 | 0.0053 | 0.0037 | 0.0049 | 0.0071 | 0.0049 | 0.0030 | 0.0045 | 0.0031 | 0.0111 |
| mean | 0,0104 | 0,0113 | 0,0123 | 0,0036 | 0,0051 | 0,0035 | 0,0050 | 0,0071 | 0,0048 | 0,0032 | 0,0046 | 0,0031 | 0,0125 |
| sd | 0,0022 | 0,0028 | 0,0039 | 0,0003 | 0,0005 | 0,0005 | 0,0005 | 0,0007 | 0,0006 | 0,0003 | 0,0005 | 0,0003 | 0,0074 |

### Summary:

Based on the analysis, statistically significant differences item functioning were shown in relation to questions: 17,18,22 (uniform) and 14 (uniform and nonuniform). Nonetheless, all pseudo R2 statistics are lower than 0.008. Further Monte Carlo simulations showed that some pseudo R2 values varied across questions, but overall they were very small under simulations that assume no DIF. For instance, the maximum pseudo R2 in Table S1 was 0.009, and thus a reasonable lower bound that would avoid Type I errors might be 0.01, which is even lower then 0.2, which interestingly corresponds to a small, non-negligible effect size (Cohen 1988).

Thus, the results indicates a lack of evidence of differential interpretation of an item across the tested groups.

## Differential item functioning in relation to gender and grade under the assumption of the Rasch model - based on the TAM package.

Supplementary table 3. DIF analyses in relation to gender based on the Rasch model (uniform DIF).

| Item ID | DIF- gender  p-value |
| --- | --- |
| Q18 | 0.003 |
| Q7 | 0.140 |
| Q8 | 0.075 |
| Q17 | 0.110 |
| Q4 | 0.032 |
| Q23 | 0.098 |
| Q9 | 0.309 |
| Q24 | 0.086 |
| Q12 | 0.002 |
| Q13 | 0.000 |
| Q5 | 0.577 |
| Q21 | 0.002 |
| Q6 | 0.000 |
| Q3 | 0.609 |
| Q16 | 0.466 |
| Q19 | 0.106 |
| Q22 | 0.020 |
| Q15 | 0.802 |
| Q2 | 0.003 |
| Q1 | 0.002 |
| Q20 | 0.206 |
| Q11 | 0.189 |
| Q10 | 0.309 |
| Q14 | 0.003 |


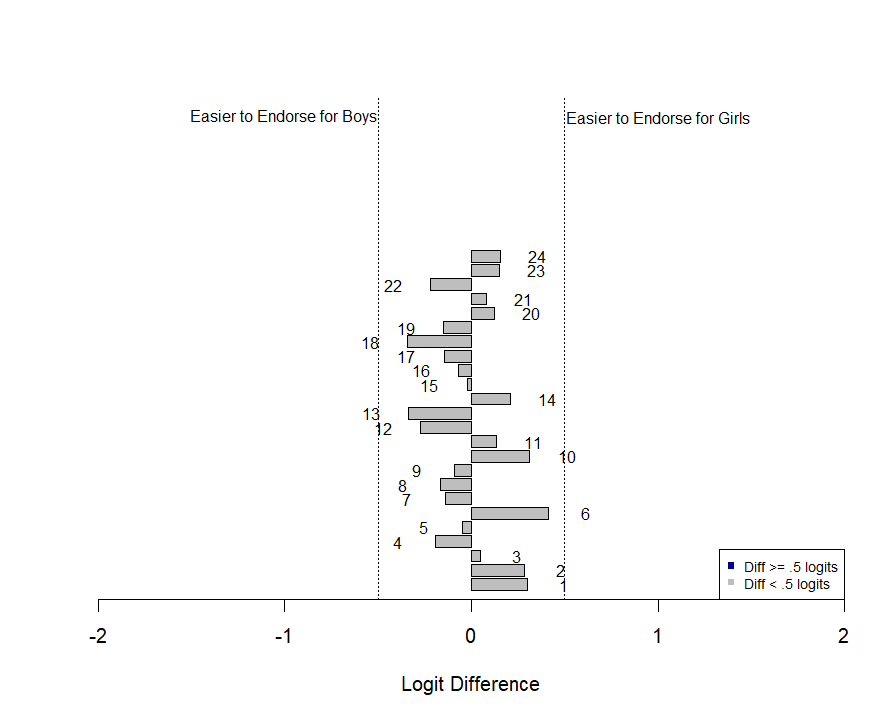


The Wald test showed that Q1, Q2, Q4, Q6, Q10, Q12, Q13, Q18 and Q22 exhibit significant DIF in relation to gender, whereas the differences between groups in the item parameters were lower than 0.5 logits and the effect size is negligible.

Supplementary table 4. DIF analyses in relation to grade based on the Rasch model (uniform DIF).

|  | Wald test (DIF - grade) | | | | | |
| --- | --- | --- | --- | --- | --- | --- |
| grades | 6-8 |  | 6-7 |  | 7-8 |  |
|  | z-statistic | p-value | z-statistic | p-value | z-statistic | p-value |
| Q1 | -0.486 | 0.627 | -0.956 | 0.339 | 0.439 | 0.661 |
| Q2 | 1.910 | 0.056 | 1.164 | 0.244 | 0.765 | 0.444 |
| Q3 | -2.367 | 0.018 | -0.271 | 0.786 | -2.080 | 0.038 |
| Q4 | -1.297 | 0.195 | -1.040 | 0.298 | -0.277 | 0.782 |
| Q5 | 2.011 | 0.044 | 0.688 | 0.492 | 1.323 | 0.186 |
| Q6 | -0.139 | 0.890 | 0.375 | 0.708 | -0.498 | 0.618 |
| Q7 | 1.369 | 0.171 | 0.276 | 0.783 | 1.092 | 0.275 |
| Q8 | -0.933 | 0.351 | -1.990 | 0.047 | 1.013 | 0.311 |
| Q9 | 1.804 | 0.071 | 1.098 | 0.272 | 0.723 | 0.469 |
| Q10 | -2.296 | 0.022 | -1.122 | 0.262 | -1.198 | 0.231 |
| Q11 | -0.263 | 0.793 | 0.068 | 0.946 | -0.324 | 0.746 |
| Q12 | 0.328 | 0.743 | 1.254 | 0.210 | -0.887 | 0.375 |
| Q13 | 2.296 | 0.022 | 2.652 | 0.008 | -0.293 | 0.770 |
| Q14 | 2.738 | 0.006 | -0.272 | 0.785 | 2.956 | 0.003 |
| Q15 | -1.645 | 0.100 | -0.477 | 0.633 | -1.168 | 0.243 |
| Q16 | -0.367 | 0.714 | -0.763 | 0.446 | 0.372 | 0.710 |
| Q17 | -3.288 | 0.001 | -1.602 | 0.109 | -1.711 | 0.087 |
| Q18 | -3.864 | 0.000 | -2.584 | 0.010 | -1.342 | 0.180 |
| Q19 | 2.134 | 0.033 | 1.366 | 0.172 | 0.791 | 0.429 |
| Q20 | -2.030 | 0.042 | -1.209 | 0.227 | -0.848 | 0.396 |
| Q21 | -0.194 | 0.846 | -1.015 | 0.310 | 0.788 | 0.431 |
| Q22 | 4.644 | 0.000 | 4.351 | 0.000 | 0.395 | 0.693 |
| Q23 | 1.138 | 0.255 | 1.602 | 0.109 | -0.425 | 0.671 |
| Q24 | -0.073 | 0.941 | -0.665 | 0.506 | 0.571 | 0.568 |

Note: Assuming the significance level as 0.05, after the Bonferroni correction is equal to 0.017. Bolded results in the above table mean statistically significant results.

The Wald test showed that Q13, Q14, Q17, Q18 and Q22 exhibit significant DIF in relation to grades, whereas the differences between groups in the item parameters were lower than 0.5 logits and the effect size is negligible, with exception of items 18 and 22 (difference between 6 and 8 grades), where the item parameters were slightly larger than 0.5.


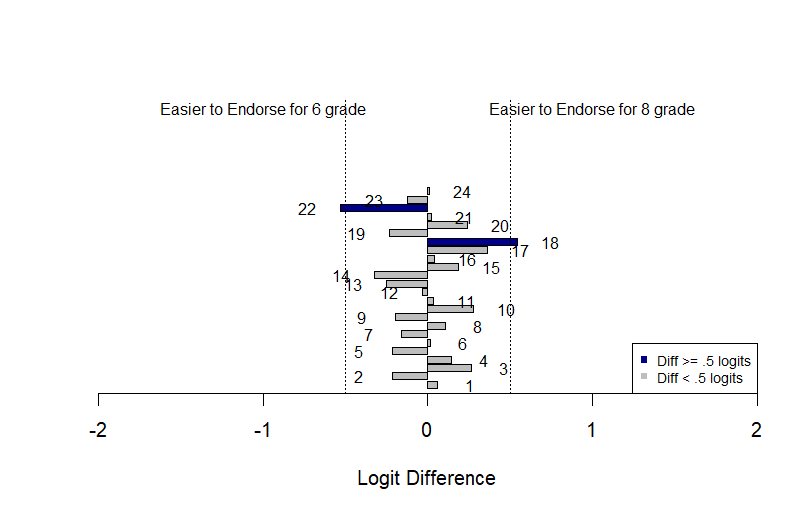


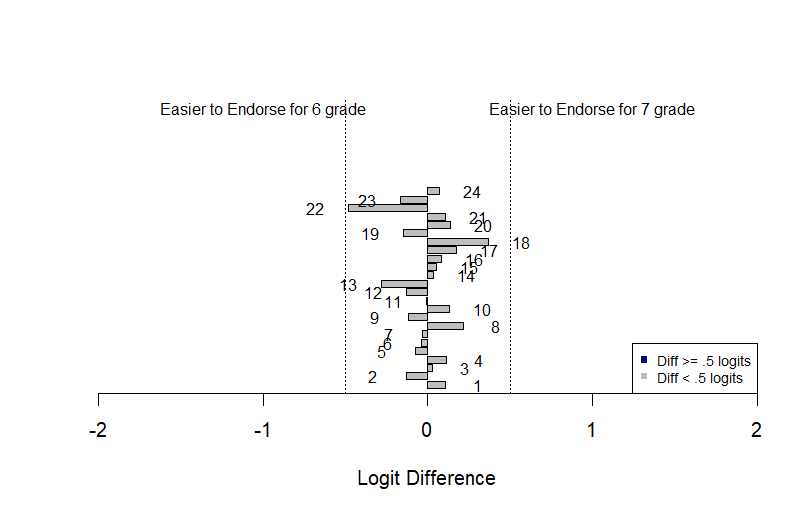


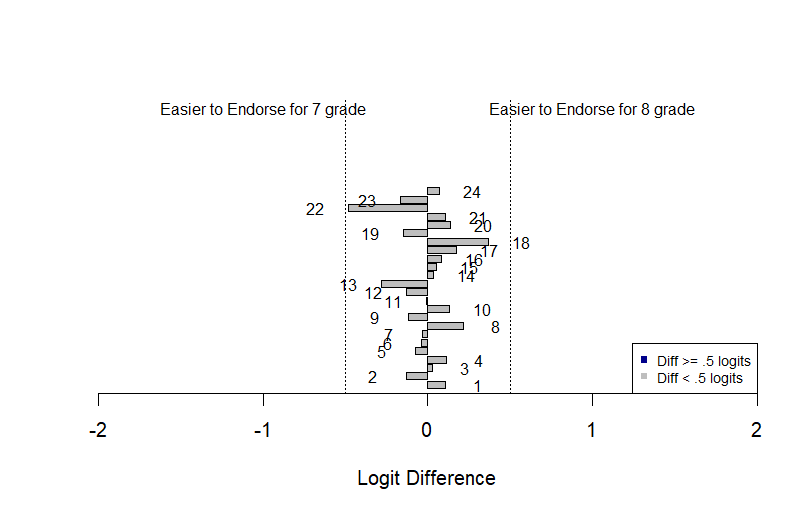

Supplement: online supplemental file 1 [file bmjopen-15-7-s001.docx]
