# Supplementary figures and images for: Measurement of critical health literacy in primary school pupils: a Polish validation of the Claim Evaluation Tools
Source: BMJ Open. 2025 Jul 17;15(7):e099994. doi: 10.1136/bmjopen-2025-099994 (PMC12273065; doi:10.1136/bmjopen-2025-099994)

Supplement 2


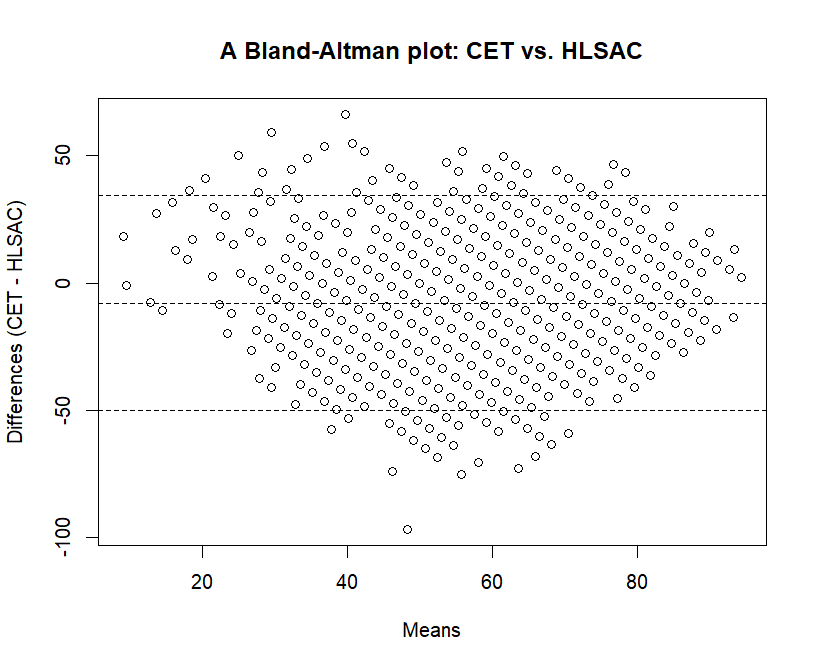


Figure S2.1. Bland- Altman plot: difference vs mean scores for the CET and HLSAC, n=2242.

Supplement: online supplemental file 2 [file bmjopen-15-7-s002.docx]
