# Supplementary material for: Measurement of critical health literacy in primary school pupils: a Polish validation of the Claim Evaluation Tools
Source: BMJ Open. 2025 Jul 17;15(7):e099994. doi: 10.1136/bmjopen-2025-099994 (PMC12273065; doi:10.1136/bmjopen-2025-099994)
Supplement: Supplementary file 3 [file bmjopen-2025-099994.reviewer_comments.pdf]

PEER REVIEW HISTORY

BMJ Open publishes all reviews undertaken for accepted manuscripts. Reviewers are asked to complete a checklist review form and are provided with free text boxes to elaborate on their assessment. These free text comments are reproduced below.

ARTICLE DETAILS

Title (Provisional)

Measurement of Critical Health Literacy in primary school pupils: a Polish validation of the Claim Evaluation Tools

Authors

Prokop-Dorner, Anna; Zawisza, Katarzyna; Świątkiewicz-Mośny, Maria; Kobla-Piłat, Aleksandra; Ożegalska-Łukasik, Natalia; Ślusarczyk, Magdalena; Potysz-Rzyman, Aleksandra; M. Bala, Malgorzata

---

VERSION 1 - REVIEW

**Reviewer**	**1**
**Name**	**Mazur, Joanna**
**Affiliation**	**University of Zielona Gora, Collegium Medicum**
**Date**	**03-Mar-2025**
**COI**	**None**

The paper is very interesting and at a high level of statistical analysis. However, there is a lack of balance between the substantive part and the description of the numerous analyses confirming the quality of the proposed adaptation. After reading the text, we know too little about the tool. We only get the information that it is good, as verified by numerous analyses. But what does the prototype look like? The word limit dictates the style of writing, but here I would argue for fewer statistics and therefore more information about the tool, which would be useful for people and centres wishing to implement it.

Major concerns

There is no main table nor supplementary one in the annex with a list of items. The third electronic annex is publicly available and can be quoted via the web address. Table 2 does not present the wording of the items only the responses. I have seen such a presentation in other papers, but I do not support it. And I have also seen other presentations of CET with both question and answer given. It would be optimal to give the 36 pre-tested items and highlight the 24 that were qualified for further analysis. There are very many references in the text to specific questions (named Qx). It is difficult to understand the main text and suppl.1 without knowing these items.

Unclear final decision on the look of the Polish version of CET. The main study tested 24 items of which 12 are key. What do the authors postulate to implement, whether 24 or 12 items. For the final suggested implementation, it is worth to clearly define the scoring rules.

In the figure 1, I suggest adding the number of students in the pilot and main survey, not just the number of schools

When describing HLSAC it is worth quoting the 5 dimensions that this tool measures (although it is a homogeneous index). One of five core dimensions is also critical HL, however understood differently. Perhaps this is why the correlation is weak. It would be interesting to see if the CET correlates more strongly with these two questions. Here it is difficult to address the content of the questions without having them given. Perhaps the CET partly refers to functional HL.

The authors describe the strengths of the survey, but do not demonstrate the limitations. One limitation is the lack of a question on the prevalence of chronic diseases and/or self-rated health. Teenagers are among the healthiest part of the population. but those in treatment may view medical services differently.

What was missing from the introduction or discussion was a reflection on who this tool is for. Is it by design for adults but also fit for young people. Is it more for patients (caregivers) but can be used in non-clinical population-based research. Whether the proposed implementation is school education towards reinforcing CHL so defined. Here, however, the problem of teacher training always arises.

Minor concerns

In the abstract N for final school survey is missing (partcipants section)

Line 39 page 7 no reference or reference [10] in wrong place

Page 8 line 36, reference [20] indicate that it is a conference presentation

Page 13 line 26 specify NUTS numer, do you mean NUTS1

The name HLSAC is not unique as there is currently HLSAC-10 and HLSAC-5.

When writing about MCQs, it is worth adding the term close-ended in brackets, as this is often confused with checklist and multiple responses analysis.

Page 13 line 51 - it would be useful to relate the pilot average to the 0-100 scale as later comparing CET and HLSAC.

Page 28 line 51 - it is better to give the averages and not just their differences.

In the appendix you have to use the same abbreviations (it is interchangeably Ix and Qx)

Highlighted as an advantage the first such adaptation in Central Europe. Maybe write in CEE countries, because however the quoted Croatia is also in (border) Central Europe although more in the southern part.

Reviewer 2

Name Finbraten, Hanne

Affiliation Inland Norway University of Applied Sciences

Date 11-Mar-2025

COI None

Thank you for the possibility of reading the interesting manuscript. The topic could be considered highly relevant. However, the manuscript has some shortcomings. I would therefore recommend editing of the text.

Introduction

For the chosen definition of health literacy, the authors have made a reference to Smith et al. (2013). However, I could not find that Smith et al (2013) defines health literacy in this way. To my understanding the chosen definition is more in line with Sørensen et al. (2012).

Critical health literacy (CHL) is mentioned for the first time at line 38, but the abbreviation is first used at line 56. The abbreviation should be presented the first time the term appears in the text. A reference to the definition of CHL is missing.

A further description of the Key Concept list should be provided. The linkage between Informed Health Choices, Key Concepts list, and the Claim Evaluation Tool (CET) should also be elaborated. To my understanding, it is not clear why the Key Concepts list is mentioned. The CET should also be more thoroughly described, especially what the concepts are about and why half of the concepts could be considered relevant for the target group. It would also be of interest to get more information about how many MCQs there are in the 12 concepts.

The sentence at line 11, page 7, lacks reference. Usually, data is tested against the unidimensional Rasch model. From my perspective, it's a bit strange to write that an instrument has an acceptable magnitude of multidimensionality. The authors have to elaborate what they mean by this statement (on page 13 the authors write that previous research indicated unidimensionality).

The knowledge gap is not clear. It has to be elaborated why this study is needed.

Materials and methods

The method section lacks information about the design of the study.

A full stop is missing at line 18, page 9.

At line 32, page 9, it is written that three MCQs were selected for an expert assessment, but at line 36 it is stated that experts were presented with 36 MCQs. This has to be explained more thoroughly. It is also stated that they adjusted 36MCQs. Does this mean that they adjusted all the MCQs? It should be described which adjustments that were made. I saw that some information about the adjustments is provided on page 16 and 17. Maybe all information about translation and cultural adaptation should be provided in the method section (and change the aim of the study accordingly)? This might make it easier to follow.

Page 10, line 15, ‘has been’ should be replaced by ‘was’. In the description of the pilot study, it lacks information about how many pupils participated (information provided in the result section). To my understanding the data collection was performed during school hours. If this is right, did the pupils have a possibility to refuse to participate?

More information is needed about the included independent variables. A table with independent variables that describes the sample characteristics would have been beneficial. It lacks a reference to HLSAC line 18, p.11. It should also be elaborated why HLSAC is used in the current study. The sentence (assess the construct) at line 28 is quite vague.

The rationale for using both CTT and Rasch modelling should be described. As MCQs could be considered dichotomous, Rasch modelling would be more appropriate than CTT. The sentence from line 47 to 53 is quite vague. It should be clarified what the authors mean here. I was also wondering why the authors use the term ‘IRT-analysis’ here, and later using the term ‘Rasch analysis’?

Data were collected in pupils from 6th to 8th grade. It would be beneficial to add information about the pupils’ age here as well, as it might differ between countries at what age a pupil is attending 6th grade.

In the national study 24 MCQs were presented to the pupils. What about the other 12? Why were these left out? I would suggest including results from the pilot study (and translation and cultural adaptation) to the methods section and focusing only on the national study in the result section. Several questions arise when reading the method section, the answers to which come first in the results section. It may be easier to follow which decisions have been made and on what basis if the authors slightly change the structure of the manuscript.

In the first paragraph on page 14 it is described the logistic function between the ability of the participants and the difficulty of the item. Here health literacy is used as an example. However, considering CET, CHL would probably be more appropriate. The paragraph also lacks reference.

The authors write that unidimensionality was verified by PCA. I suppose PCA of residuals are used to identify potential subsets? The different tests should be described more in dept for those who are not familiar with Rasch analysis.

In the description of performed Rasch analysis, I miss a description of targeting and differential item functioning.

Assessing convergent validity. CET could be considered measuring CHL while HLSAC general HL. A reference to Bland-Altman plot is missing.

Results

It would be preferable to have a table with sample characteristics of the participants in the pilot and national studies.

I wonder why the researcher chose to use CFA to verify unidimensionality. I would assume that Rasch analysis would be more appropriate for such purposes.

It would ease the reading if it was provided a reference to the table with MCQ wording when describing properties of the items, e.g. page 19.

It is unclear to me whether the results presented in Figure 2 are about the CET or the HLSAC scale. This information is not provided either in the text or in the figure.

In the text about differential item functioning (DIF) at page 27, I would recommend adding information both on which scale the DIF is evident and for which person factor. The authors also alternate between using abbreviation and not for DIF.

Line 47, page 27, do you mean scales?

Discussion

The discussion is mostly about the difficulty of the MCQs. The psychometric properties are discussed only to a small extent.

In the discussion section MCQ wording is used rather than question number. I would add question number in parenthesis. That would make it easier for the reader to follow the text.

At lines 40-43, page 31, it is stated that this study is the first to adapt and validate the CET in a Central Europe context. However, in the previous paragraph it is made a reference to a study by Hinneburg et al, in which adapted and validated a German version. The authors claim that the study has several strengths, but as far as I could see, do not reflect about potential weaknesses. I would also recommend adding some information about implications and recommendations for future use of the scale. I could not find a conclusion.

The text is relatively imprecise in several places (e.g p. 11, line 7, p. 14, line 29, p. 16). Hence, the manuscript could benefit from language editing.

VERSION 1 - AUTHOR RESPONSE

Reviewer 1	
The paper is very interesting and at a high level of statistical analysis.	We sincerely thank prof. Joanna Mazur for her careful reading of our manuscript and for the thoughtful comments. We appreciate the time and effort invested in reviewing our work. The feedback provided has been very helpful in identifying and correcting several oversights in the original version of the manuscript. As a result, we believe the revised version is significantly improved in both clarity and<br>quality.
However, there is a lack of balance between the substantive part and the description of the numerous analyses confirming the quality of the proposed adaptation. After reading the text, we know too little about the tool. We only get the information that it is good, as verified by numerous analyses. But what does the prototype look like? The word limit dictates the style of writing, but here I would argue for fewer statistics and therefore more information about the tool, which would be useful for people and centres wishing to	Thank you for the comment. Given the word limit in the initial version of the article, we had prioritized reporting the robustness of the adaptation process over the detailed description of the tool as it is available in the original publications that we referred to. In the revised version, we have added more comprehensive description of the CET, including of the structure of the CET, theoretical bases of the items, the process of development and CET usage options. We believe this adjustment improve the balance between the components of the article. Please see the changes on pages 5-7.

implement it.	
There is no main table nor supplementary one in the annex with a list of items.<br><br>Table 2 does not present the wording of the items only the responses. I have seen such a presentation in other papers, but I do not support it. And I have also seen other presentations of CET with both question and answer given. It would be optimal to give the 36 pre-tested items and highlight the 24 that were qualified for further analysis. There are very many references in the text to specific questions (named Qx). It is difficult to understand the main text and suppl.1 without knowing these items	Thank you for the comments. As we are bound by an agreement with the authors of the CET regarding the presentation of the tool, we cannot disclose the wording of the items. Below we provide Astrid Dalhgren's (the author of the validated tool) comment:<br>*Regarding publishing the test- this is something we cannot do as this is a knowledge test. Thus, publishing the multiple-choice questions would render the instrument useless for our RCT but also for any other educator or researcher wanting to use this instrument. In the same way, you will only find example questions published for the SAT-test in the USA and other similar school exams. However, this instrument, as all instrument developed from the Claim Evaluation Tools item bank is open access for teaching or research purposes on request. This is explained in the website providing access to the multiple choice questions (informedhealthchoices.org)*<br>*I hope this is acceptable to you, as it has been for other open access journals having published reports on validations of similar instruments before.*
The third electronic annex is publicly available and can be quoted via the web address.	Thank you for the comment. The optimal framework for reporting the validation process is provided by COSMIN (COSMIN- Improving the selection of outcome measurement instruments) which we refer to in the article. Unfortunately, while COSMIN offers a detailed guideline how to report the validation of a measurement tool, it does not provide a specific checklist. As BMJ Open requires the submission of a Research Reporting Guideline checklist, we uploaded the guideline that we had followed with an additional indication where in the manuscript the required information were presented.
Unclear final decision on the look of the Polish version of CET. The main study tested 24 items of which 12 are key. What do the authors postulate to implement, whether 24 or 12 items.	Thank you for your comment. The theoretical concept measured in our study (critical health literacy) has been operationalized with 12 Key Concepts. Each of them was measured by three (in pilot study) or two (in the main study) items (MCQs). Based on the analysis of data from the pilot study, we've removed 12 items (MCQs) (one item per claim). The reasons for the removal are outlined in this paragraph:<br>*Based on the analysis of 36 MCQs, we removed 12 MCQs (one per claim). Two items (Q1 and Q31) were removed due to very weak or reverse correlations with other items;*

	six items (Q9, Q11, Q20, Q22, Q28, Q34) were removed due to low factor loadings in the performed FAs (based on the whole set, after removal some items and in 3-item sets); three items (Q4, Q14, Q26) were removed based on FA and IRT results; one item (Q17) was removed due to a high percent of correct answers (90.5%).
For the final suggested implementation, it is worth to clearly define the scoring rules.	Thank you for the suggestion. The general information on the scoring of the responses presented in the paragraph on CET provides a complete description of the scoring scheme:<br>*The response to each of 36 MCQs were scored as correct (1 point) or incorrect (0 point).*<br>Since the final set of 24 items fits to the Rasch model, the final CET score as a summary of correct answers is justified. The scoring rule is to sum up the correct responses from the items. This information has been added to the manuscript:<br><br>*Scoring rule*<br>*Since the final set of 24 items fits to the Rasch model, the final CET score as a summary of correct answers is justified. The scoring rule is to sum up the correct responses from the items.*
In the figure 1, I suggest adding the number of students in the pilot and main survey, not just the number of schools	Thank you for the suggestion. We have added this information to the Figure 1.
When describing HLSAC it is worth quoting the 5 dimensions that this tool measures (although it is a homogeneous index). One of five core dimensions is also critical HL, however understood differently. Perhaps this is why the correlation is weak. It would be interesting to see if the CET correlates more strongly with these two questions. Here it is difficult to address the content of the questions without having them given. Perhaps the CET partly refers to functional HL.	Thank you for the comment. We have added the information on the HLSAC-10 dimensions:<br>*HLSAC measures subjective HL on five dimensions: theoretical knowledge, practical knowledge, critical thinking, self-awareness, citizenship (24).*<br>Thank you for the thought-provoking suggestions. We verified the correlations with the single items and found that the Spearman correlation with the items from critical thinking. They correlated positively and significantly but rather weak (0,144 for item 3 and 0,106 for item 9). The item 4 measuring practical knowledge correlated with the CET the strongest but still very weak (0.191). The other item measuring practical knowledge correlated even weaker 0,098. The strength of the correlations confirms the conceptual differences and most probable reflect the distinct nature of objective and subjective measurement.

	In the discussion we clarifies out understanding of the findings:<br>*While both of the tools assess young people's health literacy, the CET focuses on one dimension of CHL and the HLSAC studies HL more broadly by looking into five diverse dimensions. The CET objectively measures the actual knowledge and ability to recognize the health claims, while in the HLSAC-10 people self-assessed their abilities linked to health domain (10).*
The authors describe the strengths of the survey, but do not demonstrate the limitations. One limitation is the lack of a question on the prevalence of chronic diseases and/or self-rated health. Teenagers are among the healthiest part of the population. but those in treatment may view medical services differently.	Thank you for the suggestion. We agree. We have included it in the article:<br><br>*One limitation of the study is the lack of verification of respondents' health status, which made it impossible to plan a procedures for recruiting pupils participating in individualized education programs who, due to health-related conditions, could not be present in school classroom during the process of data collection. As a result, sub-group analysis between children with and without health problems could not been presented. Further studies should examine the learning and memory effect on the CET outcomes as well as explore the needs of pupils with special needs regarding their ability to navigate the CET.*
What was missing from the introduction or discussion was a reflection on who this tool is for. Is it by design for adults but also fit for young people. Is it more for patients (caregivers) but can be used in non-clinical population-based research. Whether the proposed implementation is school education towards reinforcing CHL so defined. Here, however, the problem of teacher training always arises.	Thank you for the remark. We have added the information on the target group of IHC initiative:<br>*The initiative Informed Health Choices (IHC) is based on the CHL conceptualized as the ability to critically appraise information about the effects of particular actions and to make informed health choices (9) and is connected to an IHC Key Concepts educational interventions IHC Key Concepts for children and adults (10).*<br>Moreover, in the text, we have informed that for a population sampled in this study we used the MCQs reflecting the Key Concept, as selected by authors of IHC initiative:<br>*For teaching children in primary schools, the authors indicated twelve Key Concepts selected by primary school teachers within a modified Delphi technique, as of the most relevant and easily comprehensible for primary pupils. (14).*

	Our article report on adaptation and validation of measurement tool, rather than of educational intervention/ material, therefore the notions of training teachers involved in intervention seem to us redundant.
In the abstract N for final school survey is missing (partcipants section)	Thank you for the comment. We've added the following information:<br>*We collected the data from a national sample of 2242 school pupils of 6th – 8th grades (12–15 years old) from 42 primary schools.*
Line 39 page 7 no reference or reference [10] in wrong place	Thank you for the comment. We've verified the reference.
Page 8 line 36, reference [20] indicate that it is a conference presentation	Thank you for the remark. Yes, the cited reference is a conference presentation, as the material was first presented in that and has not been published elsewhere since.
Page 13 line 26 specify NUTS numer, do you mean NUTS1	Thank you for the comment. We have made the correction.
The name HLSAC is not unique as there is currently HLSAC- 10 and HLSAC-5.	Thank you for the comment. We have corrected the name to HLSAC-10.
When writing about MCQs, it is worth adding the term close-ended in brackets, as this is often confused with checklist and multiple responses analysis.	Thank you for the suggestion. For the consistency of the description, we prefer to use the terminology presented by IHC network https://www.informedhealthchoices.org/ To make sure the abbreviation is<br>correctly understood by readers we provided the explanation of the abbreviation in the abstract, in the description of the tool and under Fig 1 and Table 4, e.g.<br><br>*The CET is an open-source batery of 188 multiple-choice questions (MCQs) measuring the ability to apply knowledge, interpret and judge the information about claims reflecting one of the Key Concepts.*
Page 13 line 51- it would be useful to relate the pilot average to the 0-100 scale as later comparing CET and HLSAC.	Thank you for the comment. The 0-100 scale was calculated only for the Bland-Altman analysis, as the two compared scales should ideally have the same range to simplify interpretation of the plot. In other cases the sum of the correct answers (1 point per correct answer) are the final scores of the scales. Thus, the averages were calculated based on the scores that were not transformed to the 0-100 range. The results are presented in Table 2, which is additionally provided.

Page 28 line 51- it is better to give the averages and not just their differences.	Thank you for the comment. The mean values of the CET and HSLAC have been added, but the difference was also presented as a part of the description of the Bland-Altman plot.
In the appendix you have to use the same abbreviations (it is interchangeably Ix and Qx)	Thank you. We unified the abbreviations.
Highlighted as an advantage the first such adaptation in Central Europe. Maybe write in CEE countries, because however the quoted Croatia is also in (border) Central Europe although more in the southern part.	Thank you for the comment. While the academic debate on the division of European regions has engaged numerous experts across various fields for decades, it does not does not yield unambiguous answers. For this reason, we took the freedom to adopt one of the common conceptualization that is the closest to our empirical insights as researchers in medical sociology, education sociology and intercultural communication and it aligns with the composition of the Visegrád Group (www.visegradgroup.eu), which includes the Czech Republic, Hungary, Poland, and Slovakia. This understanding of Central Europe reflects a shared historical, political, and socio-economic background that distinguishes these countries within the broader European context. The shared experiences determine regional similarities relevant to the topic of this study, particularly in cultural factors influencing lifestyle.<br>We clarified our understanding of the term Central Europe in the manuscript:<br><br>*To our knowledge, it is the first study to adapt the CET in Central Europe, which aligns with the Visegrád Group, comprised of Czech Republic, Poland, Slovakia and Hungary, sharing historical, social and cultural background.*
Reviewer 2	
Thank you for this interesting topic. For comments, please see attachement.<br><br><br><br>For the chosen definition of health literacy, the authors have made a reference to Smith et al. (2013). However, I could not find that Smith et al (2013) defines health literacy in this way. To my understanding the chosen definition is more in line with Sørensen et al. (2012).	Thank you to prof. Hanne Finbraten for the insightful review. We sincerely appreciate your engagement with our article. We have taken your comments and suggestion into careful consideration while preparing the revised manuscript.<br><br>Thank you for the attentive remark. The reference to Smith 2013 pertains to the sentence defining CHL later in this paragraph, while the cited definition is attributed to Sørensen 2012. We made the necessary corrections and added the reference to Sørensen.

Critical health literacy (CHL) is mentioned for the first time at line 38, but the abbreviation is first used at line 56. The abbreviation should be presented the first time the term appears in the text. A reference to the definition of CHL is missing.	Thank you for the remark. We have made the correction and added the reference.

A further description of the Key Concept list should be provided. The linkage between Informed Health Choices, Key Concepts list, and the Claim Evaluation Tool (CET) should also be elaborated. To my understanding, it is not clear why the Key Concepts list is mentioned. The CET should also be more thoroughly described, especially what the concepts are about and why half of the concepts could be considered relevant for the target group. It would also be of interest to get more information about how many MCQs there are in the 12 concepts.	Thank you for the comment. We have expanded the description of CET and more precisely explained the link between the IHC and CET and the Key Concepts within it as well as the rationales for selecting some of the items for the target group. We believe these corrections enhance the clarity and comprehensiveness of the manuscript. Please see pages [5-7] for the revised content.
The sentence at line 11, page 7, lacks reference.	The missing reference has been entered.
Usually, data is tested against the unidimensional Rasch model. From my perspective, it's a bit strange to write that an instrument has an acceptable magnitude of multidimensionality. The authors have to elaborate what they mean by this statement (on page 13 the authors write that previous research indicated unidimensionality)	Thank you for the remark. The sentence was used because the authors of the previous validation described the results in this way. Nonetheless, they assessed unidimensionality across four sets of items and achieved the results indicated that the sets were sufficiently unidimensional. Thus, the sentence has been changed to the following:<br>*The scale was found to have satisfactory reliability, unidimensionality in the data, and a high level of item difficulty.*
The knowledge gap is not clear. It has to be elaborated why this study is needed.	Thank you for the remark. We've added the following information:<br>*To our knowledge, it is the first study to adapt the CET in Central Europe, which aligns with the Visegrád Group, comprised of Czech Republic, Poland, Slovakia and Hungary, sharing historical, social and cultural background. So far the available data on health literacy in children in this region have been collected only using subjective measures (22).*

The method section lacks information about the design of the study	The information on the study design has been added:<br>*The process of the adaptation and validation comprised three phases: translation and cultural adaptation, pilot testing and cross-sectional national survey.*
At line 32, page 9, it is written that three MCQs were selected for an expert assessment, but at line 36 it is stated that experts were presented with 36 MCQs. This has to be explained more thoroughly. It is also stated that they adjusted 36MCQs.<br><br>Does this mean that they adjusted all the MCQs? It should be described which adjustments that were made. I saw that some information about the adjustments is provided on page 16 and 17. Maybe all information about translation and cultural adaptation should be provided in the method section (and change the aim of the study accordingly)? This might make it easier to follow.	Thank you. We clarified the description of the indicated passage by adding some details:<br><br>*Based on the ranking, from the translated base of MCQs three highest MCQs reflecting each of 12 Key Concepts were selected to be further assessed by experts.*<br><br>As suggested by the Reviewer 2, we have also reorganized the information on translation and adaptation by placing all the information in one section. We also worked on the clarity of the section regarding MCQs re-adjustments to make it more clear.<br><br>*Based on the expert assessment and feedback, we adjusted 36 MCQs:, namely.*<br><br>*Finally, a convenience sample of fifteen 12- to 15-year-olds filled the questionnaire and provided their comments. Based on the experts' feedback, we adjusted the wording of the scenarios and response options to the 12- to 15-year-old pupils' needs and capacities, especially in regard to plain language used to describe health problems. The adjustments included 1) names used in the scenarios to more recognizable ones in the Polish context; 2) health practices more relatable to the Polish context across all social groups (e.g. eating chocolate instead of mango; chewing gums for healthy teeth instead of eating bananas). Additionally, we excluded those MCQs which reflected practices unfamiliar in the studied context, presented content unrelatable to primary school pupils or treatments less popular these days among the youth, e.g. using herbal therapy (20). Finally, a convenience sample of fifteen 12- to 15-year-olds filled the questionnaire and provided their comments.*

In the description of the pilot study, it lacks information about how many pupils participated (information provided in the result section). To my understanding the data collection was performed during school hours. If this is right, did the pupils have a possibility to refuse to participate?	Thank you for the remark. Thank you for the comments. As the process of the validation consisted of two phases (pilot and national samples), we present the corresponding findings in the Results section.<br><br>Thank you for paying our attention to the shortcomings in the description:<br><br>*We selected pupils from 6th to 8th grade from the sampled schools (12–15-year- olds). We obtained writen informed consent both from pupils and their parents and trained moderators to collect the data. Pupils during their school hours were seated in a school computer room.*<br><br>*The data has been collected between May and June 2023 following the same procedure as in the pilot-testing survey by a team of trained interviewers according to a standardized protocol including collection of informed consent from parents and children*.
A full stop is missing at line 18, page 9. Page 10, line 15, 'has been' should be replaced by 'was'.	The corrections have been entered.
More information is needed about the included independent variables. A table with independent variables that describes the sample characteristics would have been beneficial.	Thank you for the helpful comments. A table with characteristics of the pilot study and national study samples has been provided. It is assigned as Table 2.<br><br>The reference has been added and clarified the used of HLSAC:
It lacks a reference to HLSAC line 18, p.11. It should also be elaborated why HLSAC is used in the current study. The sentence (assess the construct) at line 28 is quite vague.	As HLSAC-10 has been proven reliable on a sample of Polish teenagers, we used it to assess the construct of the CET.<br>Please note, that the convergent validity, is further described in the Methods section.
The rationale for using both CTT and Rasch modelling should be described. As MCQs could be considered dichotomous, Rasch modelling would be more appropriate than CTT.<br><br>The sentence from line 47 to 53 is quite vague. It should be clarified what the authors mean here.<br><br>I was also wondering why the authors use the term 'IRT- analysis' here, and later	We agree that Rasch modelling is more appropriate for this study, and the final decisions were based on the results of the Rasch analysis. Classical Test Theory (CTT) was used as a preliminary step — a basic analysis supporting, for example, the unidimensionality of the construct. Moreover, CTT remains one of the most commonly used techniques for scale validation. Therefore, presenting these results provides additional evidence of validity, particularly for readers who may not be familiar with Rasch analysis.

using the term ‘Rasch analysis’?	In case of pilot study and the description of methods used to choose items the manuscript was revised. In general most of decisions about choosing items for the national study was made based on the factor Analysis performed on the pilot data base. We also used Rasch analysis. Since it was preliminary analysis also Item Response Theory (IRT) technics (2PL model) were used to compare item characteristics under different assumptions.<br>In the national sample, we used only Classical Test Theory (CTT) for preliminary results and primarily relied on Rasch analysis, as described above.
Data were collected in pupils from 6th to 8th grade. It would be beneficial to add information about the pupils’ age here as well, as it might differ between countries at what age a pupil is attending 6th grade.	Thank you for the comment. The information on the studied population had been presented in the Introduction (aim formulation):<br>The aim of our study was to adapt and validate the CET to the Polish cultural context in the group of 12–15-year-old pupils of 6th – 8th grades primary schools. Our preliminary results were presented before.<br><br>We have smoothed the reading, we added the information about the age of the respondents in the methods section in two places.

In the national study 24 MCQs were presented to the pupils. What about the other 12? Why were these left out? I would suggest including results from the pilot study (and translation and cultural adaptation) to the methods section and focusing only on the national study in the result section.<br><br>Several questions arise when reading the method section, the answers to which come first in the results section. It may be easier to follow which decisions have been made and on what basis if the authors slightly change the structure of the manuscript.	Thank for the question. The pilot study was executed to help identifying the best items from among 36, so that in the national study the selected list of items was applied.<br><br>Thank you for the suggestion. We have followed it. To enhance the readability of the article, all information on translation and cultural adaptation is now presented in the Method section of the in the revised version.<br>Besides, to improve the readability of the description of the statistical analysis<br>e.g. the analysis performed, to verify whether the data fit the Rasch model some subheadings have been added.
In the first paragraph on page 14 it is described the logistic function between the ability of the participants and the difficulty of the item. Here health literacy is used as an example.<br>However, considering CET, CHL would probably be more tppropriate.	Thank you for the comment. The health literacy has been changed to CHL. The following reference has been added:<br>Tennant A Conaghan PG The Rasch measurement model in rheumatology: what is it and why use it? When should it be applied, and what should one look for in a Rasch paper? Arthritis Care Res (Hoboken). 2007;57(8):1358-

The paragraph also lacks reference.	1362. doi:10.1002/art.23108.
The authors write that unidimensionality was verified by PCA. I suppose PCA of residuals are<br>used to identify potential subsets? The different tests should be described more in dept for<br>those who are not familiar with Rasch analysis.	The paragraph has been changed as follows:<br>*Unidimensionality was verified in two ways. At first, as a the proportion of variance in the responses associated with the Rasch model. The critical value of 20% of variance explained by the Rasch model is required as evidence of unidimensionality (30). Secondly, principal component analysis (PCA) of standardized residuals (difference between observed and expected values) was done. This method verifies whether there is a patern in the residuals, which might suggest the existence of an additional dimension, or whether there is rather random noise. The value of eigenvalues of the first contrast (first PCA of residuals component) should be lower than 1.5 to support the assumption of unidimensionality.*

In the description of performed Rasch analysis, I miss a description of targeting and differential item functioning.	The description of the targeting has been added. The description of the DIF has been completed with the statistical method used. The description of the DIF analysis and methods had been included in the Supplement 1 due to the length of the manuscript.
Assessing convergent validity. CET could be considered measuring CHL while HLSAC general HL.	Thank you for the thought-provoking comment. Our perception of the tools was following: While the HLSAC measures self-reported, multi-dimensional health literacy, including a dimension of critical thinking, the CET objectively measures critical health literacy. Thus, we assumed that the tools are conceptually related; however, since they capture the concept of health literacy in different ways, we expected the correlation to be less than very strong.
A reference to Bland-Altman plot is missing.	The following reference has been added: Bland, J. M., & Altman, D. (1986).<br>Statistical methods for assessing agreement between two methods of clinical<br>measurement. *The lancet*, *327*(8476), 307-310.
Results<br>It would be preferable to have a table with sample<br>characteristics of the participants in the pilot and national studies	A table with characteristics of the pilot study and national study samples has been provided. It is assigned as Table 2.

I wonder why the researcher chose to use CFA to verify unidimensionality. I would assume that Rasch analysis would be more appropriate for such purposes.	We agree that Rasch modelling is more appropriate in this study and the final decisions were made based on the Rasch analysis. The CTT was used as the preliminary assessment. It is still most often used technique of validation of scales, thus the rationale for the presentation of the results is to provide same evidence of validity for those who are not familiar with the Rasch analysis.<br>Consistency of the results additionally support validity and reliability of the scale. Nonetheless, the unidimensionality within the context of the Rasch measurement theory was verified by the PCA of residuals.

It would ease the reading if it was provided a reference to the table with MCQ wording when describing properties of<br>the items, e.g. page 19.	The sentence “The MCQ wording is provided in table 4.", where appropriate, has been added.
It is unclear to me whether the results presented in Figure 2 are about the CET or the HLSAC scale. This information is not provided either in the text or in the figure.	The results presented in Figure 2 are about the CET. The following sentence has been added to the description of the figure 2. “The item-person map for the CET 24 item set.”
In the text about differential item functioning (DIF) at page 27, I would recommend adding information both on which scale the DIF is evident and for which person factor. The<br>authors<br>also alternate between using abbreviation and not for DIF.	According to the suggestion, the relevant information has been added. The text has been revised to consistently use the abbreviation ‘DIF’.
Line 47, page 27, do you mean scales?	Yes, it has been corrected.
Discussion<br>The discussion is mostly about the difficulty of the MCQs. The psychometric properties are discussed only to a small extent.	
In the discussion section MCQ wording is used rather than question number. I would add question number in parenthesis. That would make it easier for the reader to<br>follow the text.	Thank you for the suggestion. We added the question number to smooth the reading flow in the discussion section.

At lines 40-43, page 31, it is stated that this study is the first to adapt and validate the CET in a Central Europe context. However, in the previous paragraph it is made a reference to a study by Hinneburg et al, in which adapted and validated a German version.	Thank you for the comment. While the academic debate on the division of European regions has engaged numerous experts across various fields for decades, it does not does not yield unambiguous answers. For this reason, we took the freedom to adopt one of the common conceptualization that is the closest to our empirical insights as researchers in medical sociology, education sociology and intercultural communication and it aligns with the composition of the Visegrád Group (www.visegradgroup.eu), which includes the Czech Republic, Hungary, Poland, and Slovakia. This understanding of Central Europe reflects a shared historical, political, and socio-economic background that distinguishes

The authors claim that the study has several strengths, but as far as I could see, do not reflect about potential weaknesses. I would also recommend adding some information about implications and recommendations for future use of the scale. I could not find a conclusion.	these countries within the broader European context. The shared experiences determine regional similarities relevant to the topic of this study, particularly in cultural factors influencing lifestyle.<br>We clarified our understanding of the term Central Europe in the manuscript: *To our knowledge, it is the first study to adapt the CET in Central Europe, which aligns with the Visegrád Group, comprised of Czech Republic, Poland, Slovakia and Hungary, sharing historical, social and cultural background.*<br>Thank you for the remark. We acknowledge the omissions of the limitations and conclusions and have now included them in the manuscript:<br>*One limitation of the study is the lack of verification of respondents' health status, which made it impossible to plan a procedures for recruiting pupils participating in individualized education programs who, due to health-related conditions, could not be present in school classroom during the process of data collection. As a result, sub-group analysis between children with and without health problems could not been presented. Further studies should examine the learning and memory effect on the CET outcomes as well as explore the needs of pupils with special needs regarding their ability to navigate the CET.* The findings of this study support the validity and reliability of the adapted version of the CET. The Polish version of the CET is appropriate to use in the population of 12-15-year-olds to measure CHL. The tool can be used in educational and research settings.

The text is relatively imprecise in several places (e.g p. 11, line 7, p. 14, line 29, p. 16). Hence, the manuscript could benefit from language editing.	Thank you for the comment. We're proof-read for the clarity of the text and believe the readability of the text is improved.<br><br>*Additionally, we asked three questions regarding socio-demographics (gender, age, school grade), six regarding family situation and family's socio-economic status, one regarding self-reported reading difficulties and used the Health Literacy for School-Aged Children (HLSAC-10, 10 questions) (24).*<br>*We tested known-group validity by comparing the CET scores across gender and school grade groups. Based on other studies measuring health literacy, the following hypotheses were identified: 1) Girls score higher on the CET than boys (16); 2) respondents atending higher school grades score higher than respondents atending lower school grades (23). We used the t-test and ANOVA to compare the CET score between the groups.*

VERSION 2 - REVIEW

Reviewer **1**

Name **Mazur, Joanna**

Affiliation **University of Zielona Gora, Collegium Medicum**

Date **02-Jun-2025**

COI

The authors presented a very interesting approach to the study in HL among school-aged children. The corrections made during the review process significantly improved the quality of the manuscript. The responses to my individual comments are very detailed with changes to the text highlighted. I also read with great interest the second review and the changes made under its influence.
